# Supplementary material for: The Plasmodium heme detoxification protein functions in mitochondrial protein synthesis
Source: Nat Commun. 2026 Aug 7;17:8002. doi: 10.1038/s41467-026-76511-6 (PMC13451264; doi:10.1038/s41467-026-76511-6)
Supplement: Supplementary file 1 [file 41467_2026_76511_MOESM1_ESM.pdf]

Supplementary material

for

The *Plasmodium* heme detoxification protein functions in mitochondrial protein synthesis

Louis Sarrazin[1], Melissa R. Rosenthal[2], Joachim Kloehn[3], Tanja Ziesmann[4], Yasmin Schmitz[1], Anna-Lena Sandtmann[1], Robert Domènech-Eres[1], Katharina Scholz-Höhn[5], Coralie Boulet[3], Ute Distler[4,6], Daniel E. Goldberg[2,7] & Joachim M. Matz[1,*]

[1] Molecular Parasitology Group, Bernhard Nocht Institute for Tropical Medicine, 20359 Hamburg, Germany

[2] Division of Infectious Diseases, Washington University School of Medicine, Saint Louis, MO, United States of America

[3] Department of Microbiology and Molecular Medicine, Faculty of Medicine, University of Geneva, 1206 Geneva, Switzerland

[4] Institute for Immunology, University Medical Center Mainz, 55131 Mainz, Germany

[5] Electron Microscopy Unit, Bernhard Nocht Institute for Tropical Medicine, 20359 Hamburg, Germany

[6] Research Centre for Immunotherapy (FZI), University Medical Center Mainz, 55131 Mainz, Germany

[7] Department of Molecular Microbiology, Washington University School of Medicine, Saint Louis, MO, United States of America

* Correspondence: JMM, joachim.matz@bnitm.de

SUPPLEMENTARY FIGURES AND TABLES

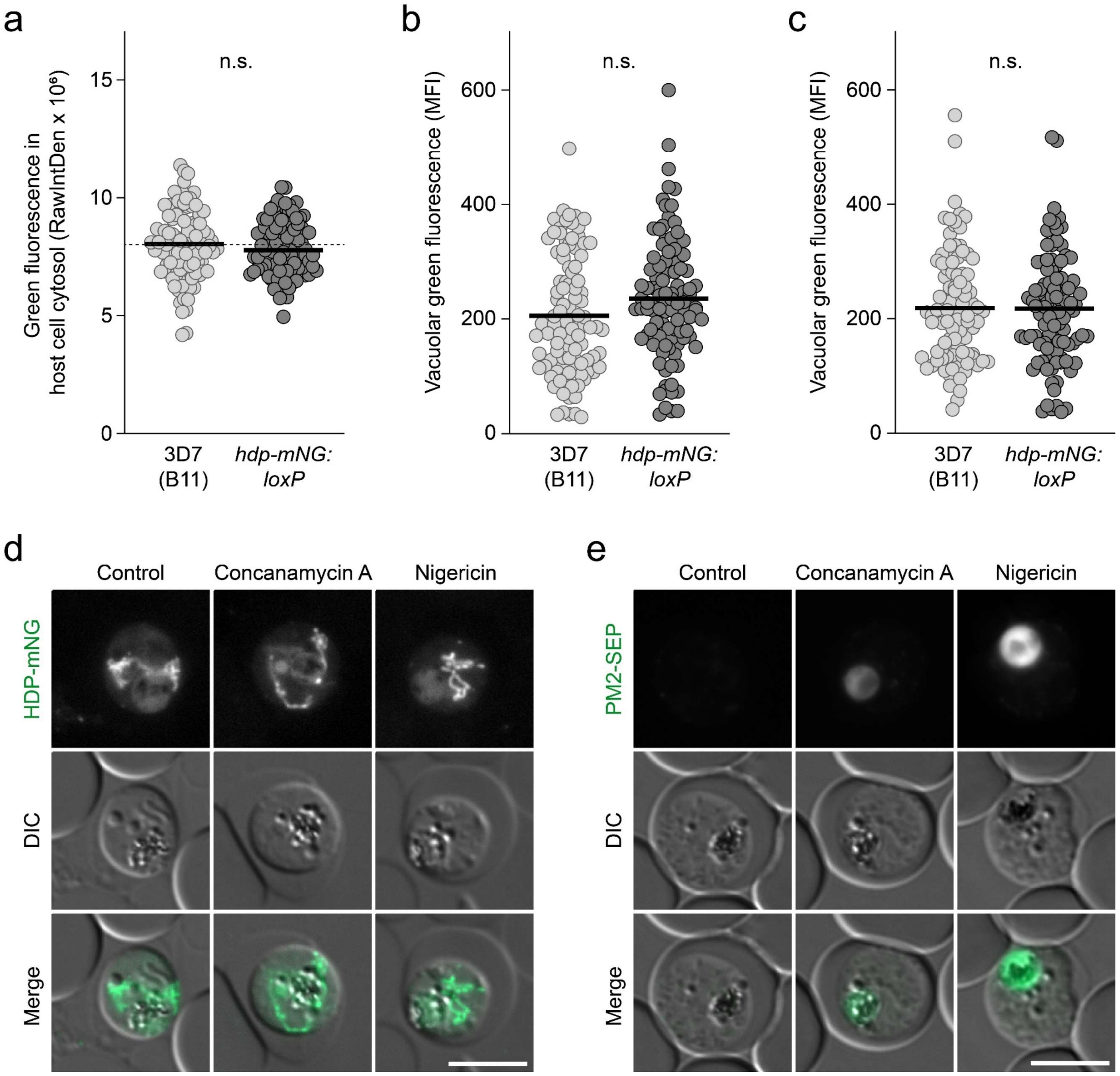

Supplementary Figure S1. **HDP-mNG is not detected in the host cell cytosol or the DV. a** Green fluorescence in the host cell compartment, quantified by live-cell microscopy of *hdp-mNG:loxP* and parental B11 parasites. The dashed line indicates the mean background intensity measured in regions devoid of cells. **b,c** Green fluorescence in the DV of *hdp-mNG:loxP* and parental B11 parasites in the presence of concanamycin A (b) or nigericin (c). n.s., non-significant, two-sided paired t-test; N = 99 parasites from 3 independent experiments. Exact P values: (a) 0.814, (b) 0.691, (c) 0.957. **d,e** Representative images of HDP-mNG (d) and PM2-SEP (e) in the presence or absence of concanamycin A or nigericin. Scale bars, 5 µm. SEP: superecliptic pHluorin; RawIntDen: raw integrated density; MFI: mean fluorescence intensity; DIC: differential interference contrast. Source data are provided as a Source Data file.

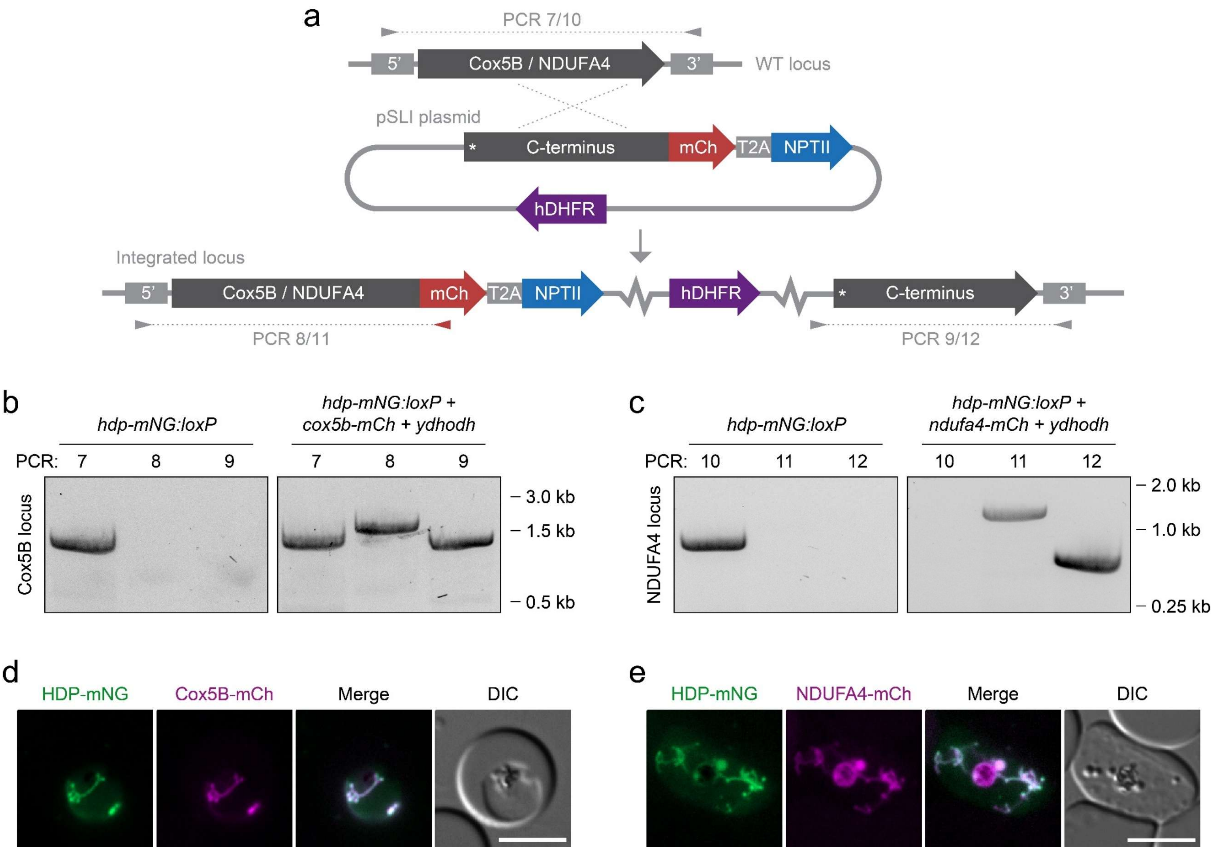

Supplementary Figure S2. **Endogenous tagging of complex IV subunits. a** Genetic strategy for endogenous tagging of Cox5B and NDUFA4 with mCherry (mCh) using the selection-linked integration system[1]. Shown are the wild-type locus, targeting plasmid, and recombined locus. Primer combinations used for diagnostic PCR are indicated. **b,c** Diagnostic PCRs as shown in a, using genomic DNA from the parental *hdp-mNG:loxP* line and the Cox5B (b) and NDUFA4 (c) tagging mutants. **d,e** Live-cell fluorescence microscopy confirms expression of mCh-tagged Cox5B (d) and NDUFA4 (e) in the tagging mutants. Although most *hdp-mNG:loxP* + *cox5b-mCh* + *ydhodh* parasites exhibited mCh fluorescence, a fraction retained the wild-type Cox5B locus despite continuous drug selection. T2A: ribosomal T2A skipping peptide; hDHFR: human dihydrofolate reductase; NPTII: neomycin phosphotransferase II; DIC: differential interference contrast. Scale bars, 5 µm. The uncropped DNA gels are provided at the end of this document.

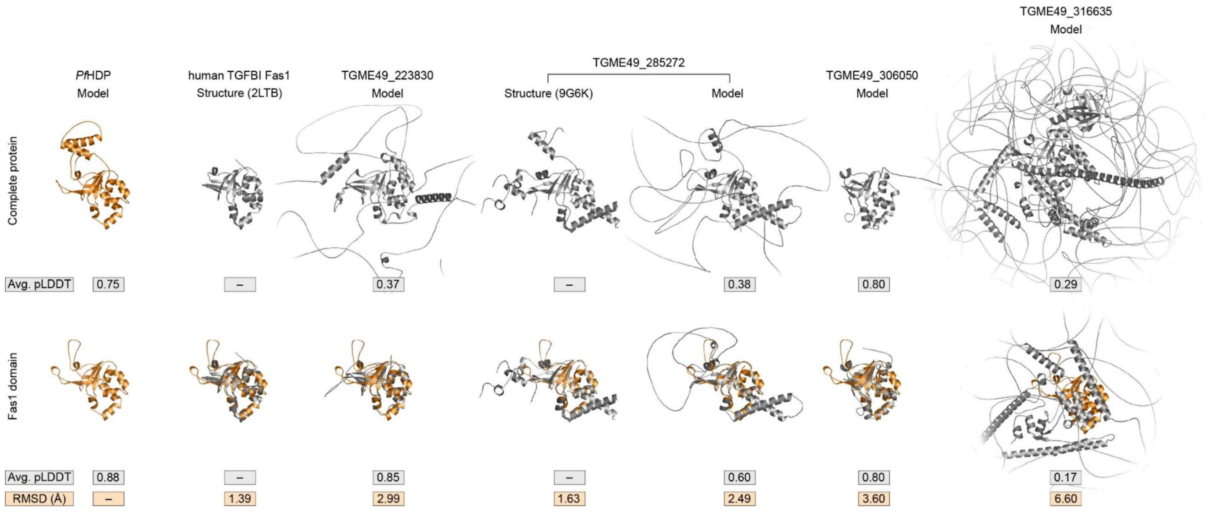

Supplementary Figure S3. **Structural alignment of HDP with Fas1-domain-containing proteins from *Toxoplasma gondii*.** Shown are AlphaFold3 models of HDP and all Fas1-domain-containing proteins encoded in the *T. gondii* genome, along with the experimentally determined structures of the mitoribosomal subunit TGME49_285272[2] and the human TGFBI Fas1 domain[3]. Whole-protein structures are displayed in the top row, while the bottom row shows alignments of Fas1 domains only. Average predicted Local Distance Difference Test (pLDDT) values for individual models and Root Mean Square Deviation (RMSD) values for the alignments are indicated.

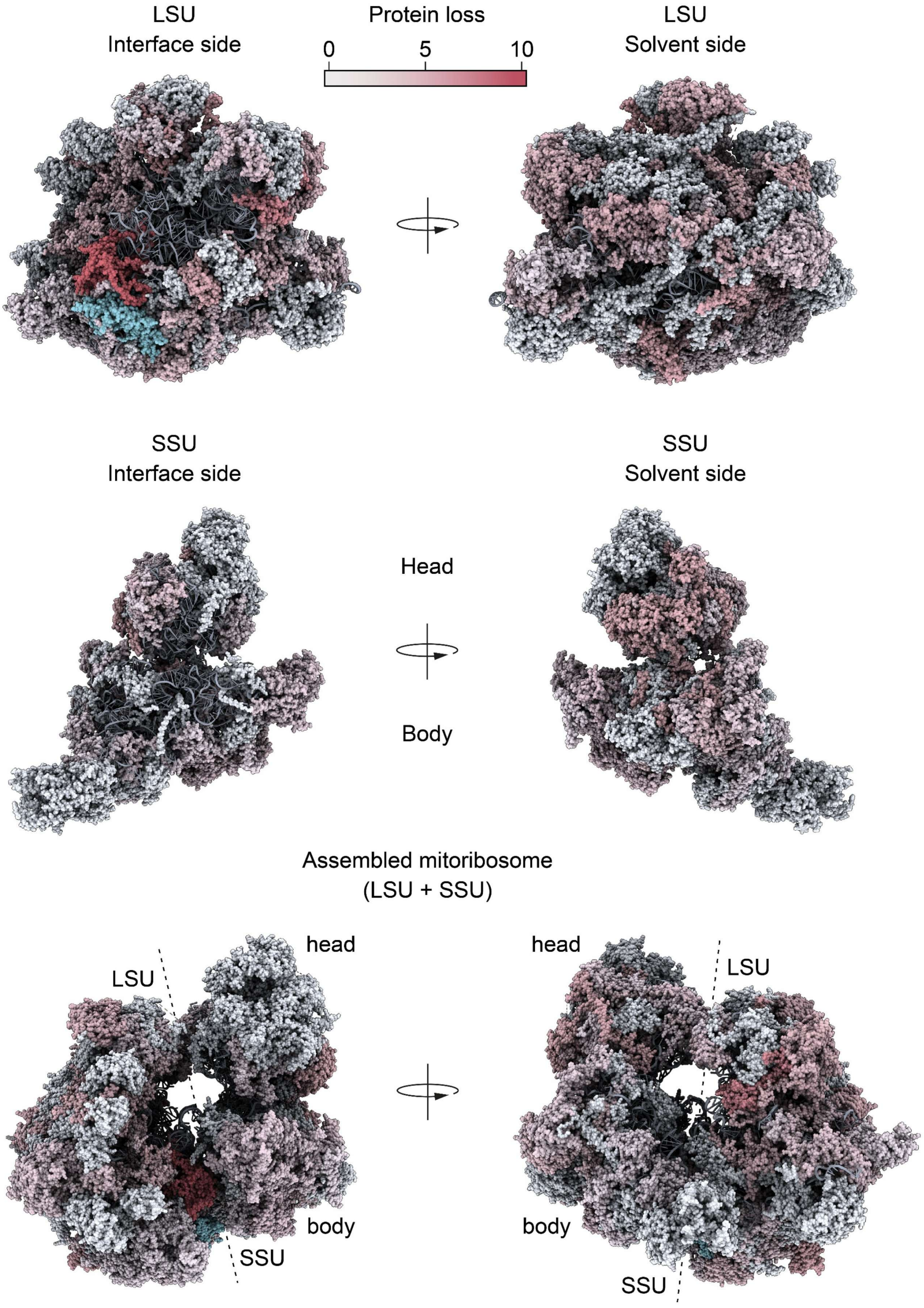

Supplementary Figure S4. **HDP-dependent reduction of *P. falciparum* mitoribosomal proteins mapped onto the *T. gondii* mitoribosome structure.** Proteins are color-coded to indicate the reduction of their respective *P. falciparum* orthologs following HDP knockout. The putative HDP ortholog TGME49_285272 is highlighted in cyan. The large (LSU) and small (SSU) ribosomal subunits are shown from both the interface and solvent sides, with additional side-views of the fully assembled mitoribosome.

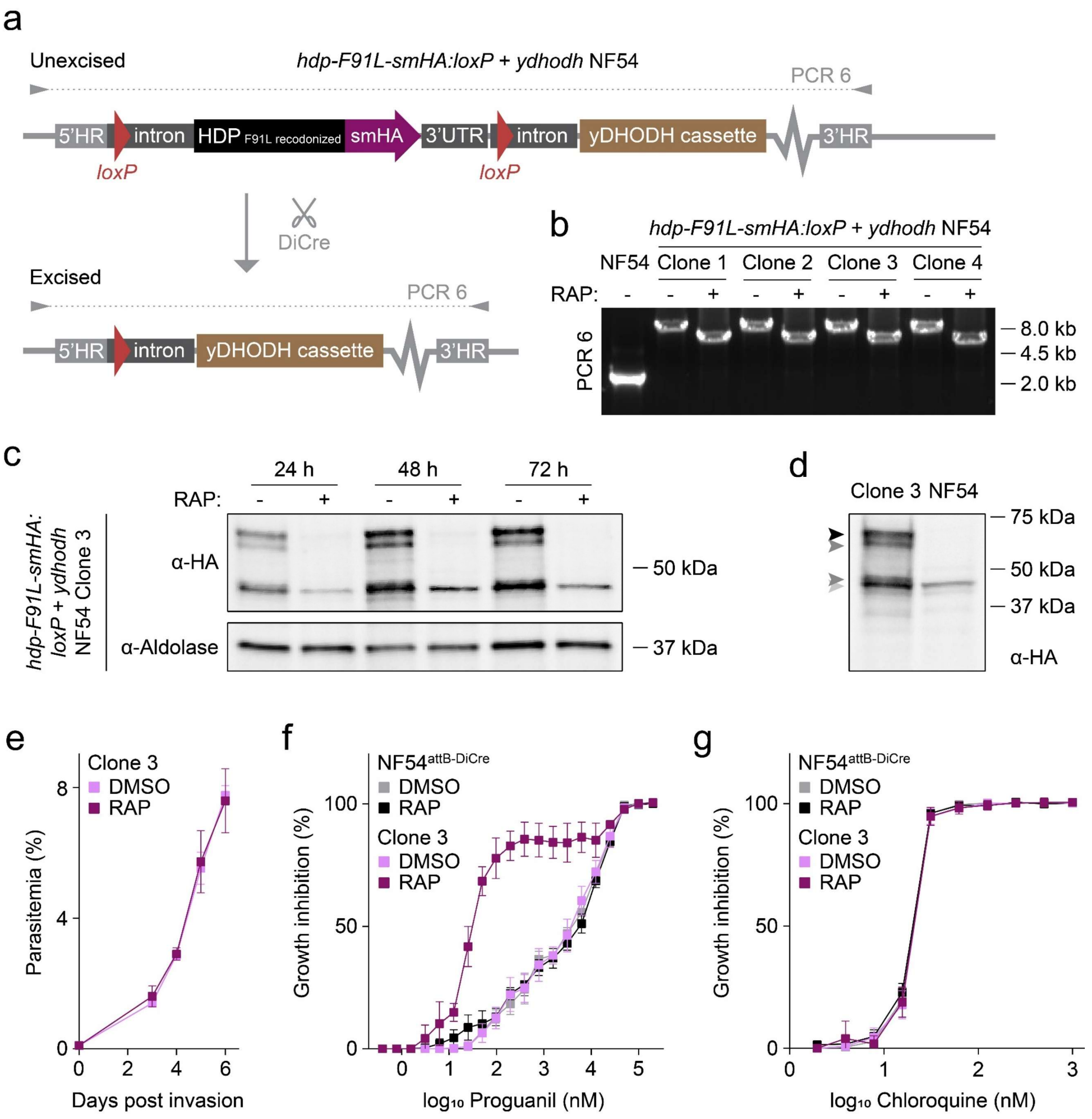

Supplementary Figure S5. **Generation and validation of an NF54-based conditional HDP knockout line metabolically rescued by yDHODH expression. a** Genetic strategy for tagging and conditional deletion of full-length HDP (variant F91L) in *P. falciparum* NF54 parasites. Shown is the recombined locus before and after DiCre-mediated excision. Primer combinations used for diagnostic PCR 6 are indicated. A yDHODH expression cassette is included to mediate metabolic rescue. Please note that the spaghetti monster hemagglutinin tag (smHA) is larger than depicted. **b-d** RAP-induced loss of HDP F91L, confirmed by (b) diagnostic PCR 6 and (c) Western blot analysis. The PCR products obtained from Clone 3 were validated by Oxford Nanopore sequencing (Supplementary Data S1). Time points for protein extraction are indicated. A non-specific protein band remains after RAP treatment and is also observed in NF54 control parasites (d). Black arrowhead: predicted size of HDP-F91L-smHA; dark grey arrowheads: possible processing products of either HDP-F91L or the smHA tag; light grey arrowhead: non-specific band. **e** *hdp-F91L-smHA:loxP* + *ydhodh* NF54 parasites exhibit normal growth in the presence of RAP. Shown are mean parasitemia +/- SEM. N = 3 independent experiments. **f,g** RAP treatment hypersensitizes *hdp-F91L-smHA:loxP* + *ydhodh* NF54 parasites to proguanil (f), but not to chloroquine (g). Comparison with parental NF54$^{attB-DiCre}$ parasites reveals no effects of the F91L amino acid substitution. Growth-response curves are shown with mean +/- SEM values. N ≥ 3 independent experiments. HR: homology region; UTR: untranslated region; yDHODH: yeast dihydroorotate dehydrogenase; DMSO: dimethyl sulfoxide; RAP: rapamycin. Source data are provided as a Source Data file. The uncropped DNA gel and blots are provided at the end of this document.

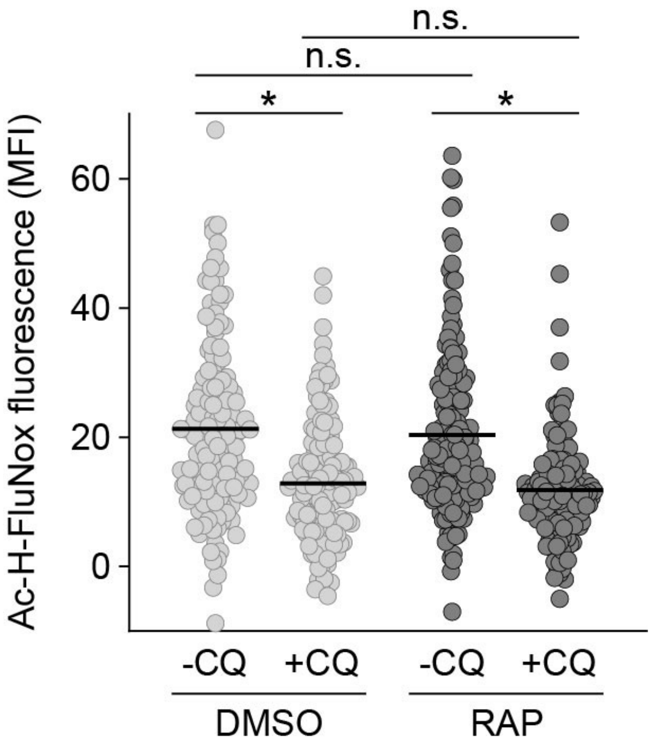

Supplementary Figure S6. **Loss of HDP does not affect levels of free cytosolic heme.** DMSO and RAP-treated *hdp-F91L-smHA:loxP* + *ydhodh* NF54 parasites were incubated with or without chloroquine (CQ), stained with the labile heme probe Ac-H-FluNox, and analyzed by live-cell microscopy to determine mean fluorescence intensity (MFI). Shown are individual and mean values. n.s., non-significant; *P<0.05, one-way ANOVA with Tukey's post hoc test; N ≥ 136 parasite from 5 independent experiments. Exact P values from top to bottom, then left to right: 0.978, 0.999, 0.045, 0.018. MFI: mean fluorescence intensity; DMSO: dimethyl sulfoxide; RAP: rapamycin. Source data are provided as a Source Data file.

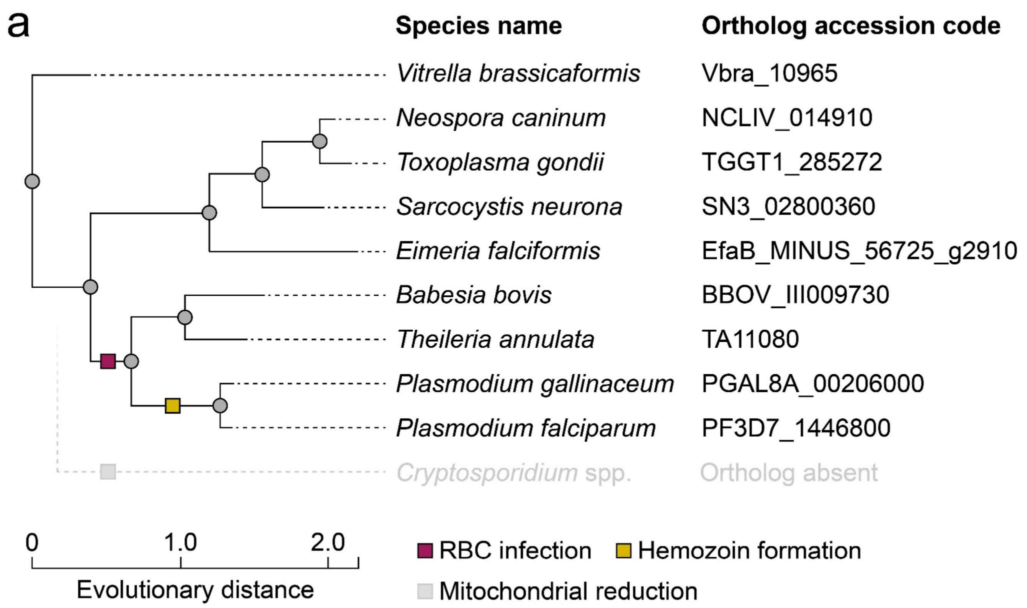

b

	V. brassicaformis	N.caninum	T. gondii	S. neurona	E. falciformis	B. bovis	T. annulata	P. gallinaceum	P. falciparum
V. brassicaformis	100								
N.caninum	28.3	100							
T. gondii	27.5	65.6	100						
S. neurona	25.0	20.5	27.2	100					
E. falciformis	26.2	30.2	29.9	27.8	100				
B. bovis	37.3	25.0	26.2	22.8	21.7	100			
T. annulata	35.2	27.8	27.8	23.0	26.7	50.3	100		
P. gallinaceum	35.7	23.0	28.1	28.0	24.7	38.3	40.6	100	
P. falciparum	37.7	23.9	27.7	27.4	22.3	38.5	40.1	82.8	100

Supplementary Figure S7. **Putative HDP orthologs are present in mitochondrion-bearing apicomplexan parasites. a** Phylogenetic tree generated from alignments of putative HDP orthologs, using NGPhylogeny.fr[4]. Gene accession codes are shown as extracted from VEuPathDB[5]. Notably, no HDP orthologs or Fas1-domain-containing proteins were detected in *Cryptosporidium* species. Relevant apomorphies are indicated on individual branches. **b** Sequence similarity matrix showing percentage identity values for pairwise comparisons among HDP orthologs. RBC: red blood cell.

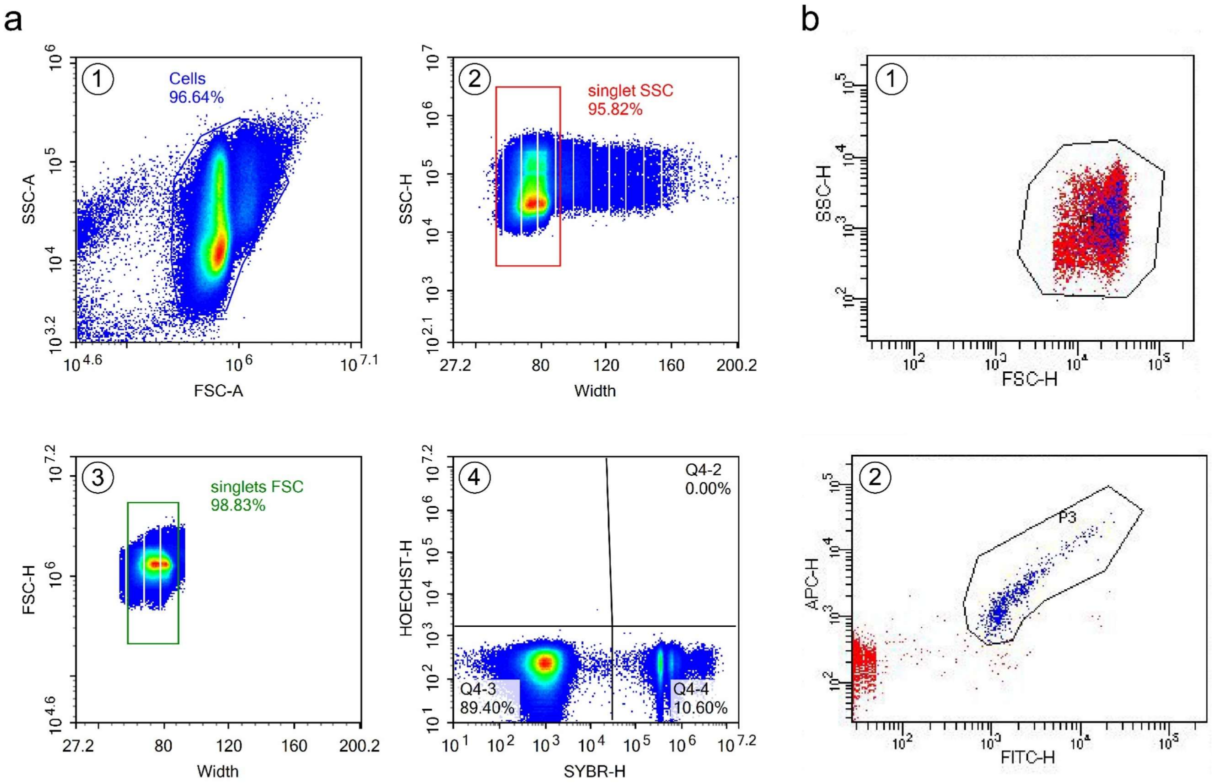

Supplementary Figure S8. **Flow cytometry-assisted identification of infected red blood cells. a** Gating strategy used for replication assays of 3D7-based parasite lines, corresponding to Figure 2e-g and Figure 3d-f. **b** Gating strategy used for growth-response assays with NF54-derived parasite lines, corresponding to Supplementary Figure S5f,g. Circled numbers indicate the order of the sequential gating steps, and individual gates are outlined. SSC: side scatter; FSC: forward scatter; A: area; H: height; SYBR/FITC channels: SYBR Green staining; APC channel: MitoTracker Deep Red staining. Gates Q4-4 and P3 identify infected erythrocytes.

Supplementary Table S1. **Oligonucleotides and repair templates used in this study.**

Template	Oligo[1]	Oligo sequence[2]	Amplicon size (bp)[3]
Diagnostic PCRs			
HDP locus 3D7	PCR 1, F	GGATTAGCCGACAAAAATTAAGG	WT 889 / Mutant –
HDP locus 3D7	PCR 1, R	ATTCATGTATTAACGAGCTGCC	WT 889 / Mutant –
HDP locus 3D7	PCR 2, F	TGAATTATCTCTCGATGGCCC	WT 2,034 / Mutant –
HDP locus 3D7	PCR 2, R	TTATTTAAGAGAGTTTGATTTATTATCTGG	WT 2,034 / Mutant –
HDP locus 3D7	PCR 3, F	GGATTAGCCGACAAAAATTAAGG	WT – / Mutant 807
HDP locus 3D7	PCR 3, R	ATAACTTCGTATAATGTATGCTATACGAAG	WT – / Mutant 807
HDP locus 3D7	PCR 4, F	AACTTCGTATAGCATACATTATACGAAG	WT – / Mutant 1,593
HDP locus 3D7	PCR 4, R	TTATTTAAGAGAGTTTGATTTATTATCTGG	WT – / Mutant 1,593
HDP locus 3D7	PCR 5, F	AATGTGTAGAATGTATTTTTCATCAG	DMSO 1,726 / RAP 507
HDP locus 3D7	PCR 5, R	TAAAACTACCTTAATGAAAAACAACATATG	DMSO 1,726 / RAP 507
HDP locus NF54	PCR 6, F	GTCTAAAGGGATTAGCCGACAAAAATTAAGGTG	DMSO 9,185 / RAP 6,498
HDP locus NF54	PCR 6, R	cctagggataacagggtaatCAGCAACTTGTTTGAAGATAATTGATTTGAAGGG	DMSO 9,185 / RAP 6,498
Cox5B locus 3D7	PCR 7, F	TATTATATCAAAATGTTAGAACCAGTCC	WT 1,457 / Mutant –
Cox5B locus 3D7	PCR 7, R	CACATGAACCAAAAACAATATGGC	WT 1,457 / Mutant –
Cox5B locus 3D7	PCR 8, F	TATTATATCAAAATGTTAGAACCAGTCC	WT – / Mutant 1,727
mCh	PCR 8, R	GTCTTCTTCTGCATTACGGGGCC	WT – / Mutant 1,727
M13R-SP6	PCR 9, F	ATGATTACGCCAAGCTATTTAGG	WT – / Mutant 1,301
Cox5B locus 3D7	PCR 9, R	CACATGAACCAAAAACAATATGGC	WT – / Mutant 1,311
NDUFA4 locus 3D7	PCR 10, F	GGGAAAAAAATGTGTAAACTTGAAAGG	WT 832 / Mutant –
NDUFA4 locus 3D7	PCR 10, R	AATATGAATATATTGTGTGCAATTATGG	WT 832 / Mutant –
NDUFA4 locus 3D7	PCR 11, F	GGGAAAAAAATGTGTAAACTTGAAAGG	WT – / Mutant 1,248
mCh	PCR 11, R	GTCTTCTTCTGCATTACGGGGCC	WT – / Mutant 1,248
M13R-SP6	PCR 12, F	ATGATTACGCCAAGCTATTTAGG	WT – / Mutant 658
NDUFA4 locus 3D7	PCR 12, R	AATATGAATATATTGTGTGCAATTATGG	WT – / Mutant 668
Cloning PCRs			
mCh	F	aaaaaaacgcgtgctagaggtgctgctgctggtgctggaggtgcaggtagacgtacgATGGTGAGCAAGGGCG	777
mCh	R	aaaatagtcgacCTTGTACAGCTCGTCCATG	777
mNG	F	aaatttgatatcggagcaggtgcaggtATGGTAAGTAAGGGAGAAGAAGAC	747
mNG	R	tttttagatatcCTTATACAACTCATCCATTCCCATTAC	747
Cox5B (3D7) CT	F	aattatgcggccgctaaCCGAAGCAACTAAACTTTTGTGGA	1,063
Cox5B (3D7) CT	R	atatttacgcgtTGTGTTTGGTTTTTGGTTTCCCCATTTTCC	1,063
NDUFA4 (3D7) CT	F	attaaaagcggccgctaaCTCATATTTCAGAACGCACAACAT	567
NDUFA4 (3D7) CT	R	atatttacgcgtAACTCGCATGTTTTCTGGTTTTTG	567
HDP (3D7)	F	aaactactcgagATGAAAAATAGATTTTATTATAATTTGATAATTAAAAGATTATATACAC	654$_{cDNA}$
HDP (3D7)	R	aaattcggtacctcctgctcctgctccAAAAATGATGGGCTTATCTACTATATGC	654$_{cDNA}$
HDP (3D7) NTD	F	aaactactcgagATGAAAAATAGATTTTATTATAATTTGATAATTAAAAGATTATATACAC	204
HDP (3D7) NTD	R	ttttttggtacctcctgctcctgctccTATTAAATTAATAATTGTCCTTTTTACAG	204
HDP (NF54) 5’HR	F	cttcaaacaagttgctgggcgcgccTGAACTATGATTTCTACATACATATAATATTC	478
HDP (NF54) 5’HR	R	gtatattatttttttatttacACTGATGAAAAATACATTCTACACATTTATACAG	478
HDP (NF54) 3’HR	F	ggtgacactatagaactcgagGTTTTAAAACATCAACAATTTATTACATAC	904
HDP (NF54) 3’HR	R	gtagaaatcatagttcaggcgcgccCAGCAACTTGTTTGAAGATAATTGATTTGAAGGG	904
LoxP	F	gtagaatgtatttttcatcagtGTAAATAAAAAAAATAATATAC	152
LoxP	R	caagttgtaatagaatctatttttcatCTAAAAGAATATAAAA	152
HDP F91L recod	F	ttttatattcttttagATGAAAAATAGATTCTATTACAACTTG	661
HDP F91L recod	R	cacatcataagggtacattcctccacttccAAAGATGATGGGCTTGTC	661
smHA	F	gcccatcatctttggaagtggaggaATGTACCCTTATGATGTGCCCGATTATGCTG	1,100
smHA	R	attatataactcgacgcggccgTTATTAAGCGTAGTCCGGGACATCGTACGGG	1,100
Guide plasmid construction[4]			
HDP (3D7)	F	attgTCATCCTACAGGTAACAACA	
HDP (3D7)	R	aaacTGTTGTTACCTGTAGGATGA	
HDP (NF54) #1	F	taagtatataatattCTATATGCACAACTCCATTGgttttagagctagaa	
HDP (NF54) #1	R	ttctagctctaaaacCAATGGAGTTGTGCATATAGaatattatatactta	
HDP (NF54) #2	F	taagtatataatattACATATGTTGTTTTTCATTAgttttagagctagaa	
HDP (NF54) #2	R	ttctagctctaaaacTAATGAAAAACAACATATGTaatattatatactta	

Repair template sequences[5]

hdp-mNG:loxP (3D7)

AATGATTGTCATTTATTTTCATAATAATTCTTTCTCAAATGTAAGAAAAGAAAATGATAACTTTTGTTAAACATAAAACATTTCTTTTTTTTTCTTTTTTTTTTGGTCTAAA
TAATATATATTGTATTATATTGTATTACATTATATATCTGTATAAATGTGTAGAATGTATTTTTCATCAGTATGAAAAATAGATTTTATTATAATTTGATAATTAAAAGATT
ATATACACGAAGTGGCGGTTTAAGAAAACCTCAAAAGGTAACCAACGACCCAGAAAGTATAAATAGAAAAGTATATTGGTGTTTTGAACATAAGCCTGTAAAAAGGA
CAATTATTAATTTAATATATTCACATAACGAACTCAAGATATTTTCTAATCTGTTAAATCATCCTACAGGTAAATAAAAAAAATAATATACAATAACTTCGTATAGCATAC
ATTATACGAAGTTATTATATATGTATATATATATATATTTATATATTTTATATTCTTTTAGTTGGCTCATCATTGATACACGAGCTTTCATTAGACGGTCCTTACACTGCA
TTTTTCCCAAGTAACGAAGCCATGCAGTTAATAAACATAGAGTCATTCAACAAGTTATACAATGATGAAAACAAGTTGTCAGAGTTCGTTCTTAATCACGTGACCAAG
GAATACTGGTTGTACCGTGATTTATACGGATCTTCTTATCAGCCTTGGCTTATGTACAATGAAAAAAGGGAGGCTCCAGAGAAATTGAGGAACTTACTTAATAACGA
CTTGATTGTAAAAATAGAAGGTGAATTCAAGCATTGTAACCACTCTATATATTTGAATGGTAGTAAGATTATTAGACCTAACATGAAGTGCCACAATGGAGTGGTACA
CATTGTTGATAAGCCTATAATATTTGTTATCGGAGCAGGTGCAGGTATGGTAAGTAAGGGAGAAGAAGACAACATGGCATCATTGCCAGCCACCCACGAGTTGCAT
ATTTTCGGTTCTATTAACGGAGTGGACTTTGATATGGTTGGTCAGGGTACAGGAAATCCTAATGATGGTTATGAGGAGCTTAATTTGAAATCTACTAAAGGTGACTT
GCAGTTTTCACCTTGGATACTTGTTCCACATATAGGTTACGGTTTTCACCAGTACCTTCCATATCCAGATGGAATGTCTCCATTCCAGGCTGCTATGGTGGATGGAA
GTGGTTATCAAGTACATAGGACCATGCAGTTTGAGGACGGTGCATCTTTAACCGTTAACTATAGATATACCTATGAAGGATCTCACATTAAAGGTGAAGCCCAAGTG
AAAGGTACCGGATTTCCTGCTGACGGTCCAGTGATGACTAACAGTTTAACTGCCGCAGACTGGTGCCGTTCTAAAAAGACATATCCAAACGATAAGACTATAATTTC
TACCTTTAAATGGTCATACACAACAGGAAATGGAAAACGTTATAGATCAACTGCAAGGACTACTTATACATTCGCTAAACCAATGGCCGCCAATTATCTTAAAAATCA
ACCTATGTATGTTTTCCGTAAAACTGAATTAAAGCACTCAAAGACAGAACTTAATTTTAAGGAATGGCAAAAAGCATTTACTGACGTAATGGGAATGGATGAGTTGTA
TAAGGATAACTAAAATATAACTTCGTATAGCATACATTATACGAAGTTATAAAACAACACAGATAAATTAACACACACACACATATATATATATATATATATATATATTAATT
TATTTGTTATATATTTATGATTTATTTTAAAAATATTATTTTTTTTTATTTTTATGGGAAAAATGAAGGATATTCCTTTTTCATTTTAAAAATATGTTTTTACTTGAAAAATAT
TACATATGTTGTTTTTCATTAAGGTAGTTTTAAAACATCAACAATTTATTACATACATATATATATTATAAAAGTGTAATATAATTGTTCTTTTAAAATAATAAATAAATTA
TAAAAAAAATATAAAAATATAAAATATAAGGATAACCTAGTACATATTATATGAAACAAAAAAAAAAAAAAAAAAAAAAAAAAATGGTATATTAAAATTTTTTTTTTTACAAA
AAA

hdp-F91L-smHA:loxP + ydhodh (NF54)

TGAACTATGATTTCTACATACATATAATATTCCTATAAATTTGGAATGACACAATTATGCAATATATATATATATATATATATATATATATATATATATAATATTTTAATTAAG
TAAAAAAATAAGAATTAACGTATTAAAAGAAAACAATCATTAAGTTGTATTTTTTTTTTTTAACATATTTTAATTAATAATGTAATATAAATAAATAAAGTAAATTATATATT
ATAATTTTTCACTTTTTATGTCAAAATGATTGTCATTTATTTTCATAATAATTCTTTCTCAAATGTAAGAAAAGAAAATGATAACTTTTGTTAAACATAAAACATTTCTTTT
TTTTTCTTTTTTTTTTGGTCTAAATAATATATATTGTATTATATTGTATTACATTATATATCTGTATAAATGTGTAGAATGTATTTTTCATCAGTGTAAATAAAAAAAATAAT
ATACAATAACTTCGTATAGCATACATTATACGAAGTTATTATATATGTATATATATATATATTTATATATTTTATATTCTTTTAGATGAAAAATAGATTCTATTACAACTTG
ATTATAAAAAGATTATACACTCGTAGTGGGGGATTAAGAAAGCCACAAAAAGTTACCAACGACCCTGAAAGTATTAATAGAAAGGTTTATTGGTGCTTTGAACATAAA
CCAGTAAAAAGAACAATAATTAACTTAATTTATTCACATAACGAACTGAAAATTTTTTCTAATCTCTTAAATCACCCAACAGTTGGCAGTTCCTTAATACATGAGTTATC
TCTGGATGGGCCTTATACTGCTTTTCTTCCGTCCAACGAAGCGATGCAATTAATTAATATTGAAAGTTTTAATAAATTGTATAACGACGAAAATAAATTGTCAGAATTT
GTTTTAAATCATGTTACGAAGGAATATTGGCTCTATAGAGATTTGTATGGATCATCATACCAACCCTGGTTAATGTACAACGAAAAAAGAGAAGCACCAGAGAAATTG
AGAAATTTGTTAAATAATGACTTAATTGTAAAAATAGAGGGGGAATTCAAACATTGCAATCATTCAATTTATTTAAATGGGTCAAAAATAATTAGACCTAATATGAAATG
CCATAACGGTGTAGTCCATATTGTAGACAAGCCCATCATCTTTGGAAGTGGAGGAATGTACCCTTATGATGTGCCCGATTATGCTGGCTACCCTTATGATGTTCCTG

ATTACGCCGGATATCCGTATGACGTGCCAGACTACGCGGGAGGTGTGAGCAAGGGAGAGGAGTTGTTTACCGGCGTGGTCCCCATACTGGTGGAGTTGGATGGC
GACGTTAATGGACATAAATTCTCGGTCCGCGGCGAGGGAGAGGGAGACGCCACCAATGGCAAGCTGACGCTTAAGTTCATTTGCACGACTGGAAAATTGCCTGTC
CCCTGGCCGACCCTGGTCACCACACTGGGCGGAGGAGTGCAGTGCTTCTCCCGTTACCCAGACCACATGAAGCAGCACGATTTCTTCAAGAGCGCAATGCCGGA
GGGATACGTGCAAGAACGGACTATTTCCTTCAAGGATGACGGAACCTATAAGACCCGTGCGGAGGTCAAATTCGAGGGTGACACCCTGGTGAACCGAATTGAACT
CAAAGGAATCGATTTCAAGGAGGATGGAAATATTCTGGGTCACAAGCTGGAATACAACTTCAACAGCCATAATGTGTACATTACGGCTGATAAGCAGAAGAACGGC
ATTAAGGCCAATTTCAAGATCCGCCACAACGTTGAGGGTGGATATCCCTACGACGTGCCCGATTATGCGGGCGGATATCCGTATGATGTTCCAGATTATGCTGGTG
GTGGAGGCTATCCCTATGATGTCCCCGACTACGCCGGAGGTTACCCATACGACGTGCCCGACTACGCTGGAGGCGATGGCAGCGTGCAGCTGGCAGATCATTAT
CAACAGAATACCCCCATAGGTGATGGCCCCGTTCTGCTTCCAGATAATCACTACCTTTCCACCCAGAGCGTGCTTTCGAAAGACCCGAACGAAAAGCGTGATCACA
TGGTCCTGCTGGAGTTTGTGACCGCGGCTGGAATCACCCTGGGTATGGACGAACTCTACAAGGGTGGCTACCCCTACGATGTGCCGGATTACGCTGGATATCCGT
ATGACGTACCGGACTATGCCGGTTACCCGTACGATGTCCCGGACTACGCTTAATAACGGCCGCGTCGAGTTATATAATATATTTATGTACTCACAATGGGGTCTAC
AAAAAAAAAAAAATAAAGATATATATATATATATATATATATATATATATATATATATATATATATTATCTTTCATTTTATCATCACCCCAATATACATATATATATTTTTATATTT
TTATATATTTATATTTTTCTTTTATTTATCCATAAAAGAAAATTAAATATAAATTTATACCTTTTATTTGAATTACCATTTGTATAAAATATAAAAAAAAAAAAAAAAATAATA
AAAAGAAGAAAAAACGAAAAAAGTAATATTTAAATAATAAAGATCAATGTACATAATAAAAAAAAAAAAAAAAAAAAAAAATTAAAAATTAAAAAATTAAACATTGAAATAAT
TTCATATACACACATATACATACATACATATATATATATATATATATATATATTTATACATTTATGCATATAAAATATATTAACAACTTATGTGTATTCATATATATTTTT
TTACATATGTATTGAAAGACTTTACTGAGACATGAAATATCTTATAACCAAACGTTTATAAATTCTAATTATATGTAAAGCATAATATAGCTTTTGTCTAATTTAATTCCT
TAAAACGTATGTATTAAAAATATAGTAAGATGGGGAAATTATAATTTTTTTTTTAAAGGTTTTTCTTTGATACAGTAAAAAAAAAAAAATAAAAAAAAATAATAATAATAAA
AAAATAAAAAAACAAAAAAAAATAAAATAATTATAAAGATATATATAATTCCTCCCAAATACAAATGATTAAGTTTAAAAATTAATAAATAAATCAAAAAAAAATTTAATA
ATAATTGTATTATTTTAAGTGTAGTTAATTCATCAAATAAGGTAGCATTATGAAGCTTGCATGCCTGCAGGGTAAATAAAAAAAATAATATACAATAACTTCGTATAGC
ATACATTATACGAAGTTATTATATATGTATATATATATATATTTATATATTTTATATTCTTTTAGAATTCTAGATTTAATAAATATGTTCTTATATATAATGAGAAATAAATA
TTTAACATATGTTTAAAAAGAAAAATTTAAGATTTACATGATTAGGAATAAAAATATTAAAAGCTATATATTAATGTTGTACTTTATGTTCATATTGTAAAAGATAAATAT
AATTTTTATTAAAATTTATATTTATTATTATTATAAGTTCTTAGAAAATACATTTTGTTTTAAAATAATACTATAAAAAGAACAATAATAAAAATAAAATACAATTAAAACAT
AATTAAAAAAGAAAAATACAGACAGTAAAAAAAATCGCTATCCCATAAATTACAAAACATGAATTAATAAACATATATTGTTTATTAATAAAATTGTAAGTTATTTTTATA
TATACATAATAAATTAAAGATACTGAAACAATAATTTTAAATGTAAATAAATGAATAATGATATGTTTTATATGATTCATTATTCTATATTTATAAGGAAGATTACAAAAA
AAAAATTCATATGTATTTTTTTTGTAATTTCTGTGTTTATGTTTTTATTTTTATAATATTTTTAATCTATTATTAAATAAGCTTTTAAATGCTGTTCAACTTCCCACGGAAC
TGATCTATGGATGTATAACCCTTAGCTTCCATTATGTCTTTTAATTCTTTTTCGATACGTTCAAAAATCTTGACGCCCTCTTTTTGTAATTCTGTACCAATCTGTAGCAT
AGAGGCACCACATAGAAGATGTTCAAATGCATCCTTACCGGACTTAATTCCACCTGTACCGATAACTTTGATTTCAGGTCTCAAACGAGTGTAAAATGCACGAACAT
TGGCGAGCGCGGTTGGCTTAACATATTCACCTCCAATACCCCCGAAACCATTCTTTGGCTTCACTACTACACTCTCCTTCTCCACATCAATGAAAAGACCATTTCCT
ATACTATTGATAGAGTTGACATAAGCTAATGGGAACTCGTTCAATATTTTTGCCATGATATCAAAATGGGCAAAATCAAAATAAGGAGGCAACTTGACACCAAGAGGT
TTTTTGAAAAAGGCAAAAACCTTTTCCAAGGTTTCCTTTGTCAAGTCAAAGTCATAAGCAACTTGTGGTTTCCCAGGCACATTAGGACAAGACAAGTTTAACTCGGTA
ATACCGTTGAATTCGCTATCTTGGATTTTCCTCAACAAATTTAAATTTTCATCAATGCTCATACCAGCAACTGAGAAGAAAATAGCAGGTGCATCAGGATAATTCTTTT
GACGGTTTAATACATAGGACAAATAGTAGTCGATACCTTCGTTTGGTAAACCCATGGAGTTGATACTGCCTAGAGGGACAGAAATGTAACGTGGTTCAGGGTTACC
TTCTCTTTCTAAGGTTGTAGCACTCTTTGTAATGAATGCGCCAGCTTTAGAGTTTGCTAATTCATCTAATTCTTGTGTAGTCATGCAATGAACACCGGATGCATTCATA
AATGGGTTTTCATAGGTATTGTTCAAGAACTTGGTAGTTAAACTGGCTGTCATGGATCCTGATATATTTCTATTAGGTATTTATTATTATAAAATATAAATCTTGAATGA
TAATAAATAAAATATTAGTTATTCCTTTTCTAGTTTAAAATATACATATTATAAATATATATATATATATATATATATTTTTATTGTGACAAGAATATATAATTATAAATTATAT
TATTTATTTTTGTATTTTTTTTTTTTTTTTTTTTTTTTTCTTTTTTTGTTTTATTTTTCTTTTTTTTTATAAATATTATTTTTTTCTTTTATCATGCACATTGGAATAATACATTA
ATATATATATATATATTATATTATACATATATTGAATAATGTTTATAAAAAATGCATAACTTATATGAATATAATTTTTTTTAAATATGACAAAAAGAAAAAAAAAAAAAAAC
CAAAAAAAATTAAAATTGAAATGAAATATATAAATATATTATTTATATATATATTATACATTGTTTAATACTACTACATGTATATATATATATATTATATATATATATATATATCAA
TTTTTTCAAAAATAAATTAATATAAAAAGAGGGGAAAAAAAAAAAAAAAAAAAAAAAAAAAGATAATTAAGTAAGCATTTAAAAATATATAAATTGATAATATATATAAAATTAA
TCACATATAAACTAATATAATTTATAAAATAAGGAAAATAAAATATTACCATAAAATAAAAATAAAAATAAAAAAAAAAAAAAAAAAACACCTTTTTTTATATATATATTAATAT
ATAATTATCTCTTAGAAAAAATATTGTATAATTATATATGTAATGATTTATATAAAAAAATAAAATTATACAAGTATATATTTTGTTTCTATAAATTGATATCTTAATTATTT
ATTATTAGAAATAGATATTTTTATAATAAACCAATAGATAAAATTTGTAGAGAAAAAAAAAATAAAAATAAAAATAAAAATAATATAATATATATAATAAAATAAAATAATATTA
TATAAATATATTTTAATTTTTTTTACAAAATGGTTA --- (*E. coli* backbone) --- GTTTTAAAACATCAACAATTTATTACATACATATATATATATTATAAAAGTGTAATA
TAATTGTTCTTTTAAAATAATAAATAAATTATAAAAAAAATATAAAAATATAAAATATAAGGATAACCTAGTACATATTATATGAAACAAAAAAAAAAAAAAAAAAAAAA
AATGGTATATTAAAATTTTTTTTTTACAAAAAAATATATGATATATGGTAAAATAAATAATATATTGGATAAGATGAAAAAATTAAAATATTCACAAAAATGTATATAATA
TATATATGTCATATATTATAACCATGTGTAACAAAATCTTATACAATATATAATACAAAAACACAAATATTATATATATATATATATATATATACCATATGGTCACATTTATAT
GTTTCATTTATTTATATGTTTATATGTTTTTTCCTTTTTGATCTTATATACCTCATATTTTGTTACCTATCGTTTTATAATATTCCTTTAAAAATACATTCGGAGCAAATGC
ATTCTTATTTCTTACAAATGTTATTTCAAAAATATTGGACCACAATTTTCCGGTAACTAGCAAGGTGGGTAATTCATCTTGCCTACTTTCGGGTCTTATGGCAATTCCA
TTAAGTACGGTCTCTAACTTGTTCTTAATTTGTTTCTACAAAAATGAAATAAAATAAATAAATAAATAAATATGTAAATAGTATGCATAGTAAATATATCATAAATATATA
TATATATATTTATTTATTTATCTATCTATTCATTTATATGTTTGTTATATAAATTATTGTATCCTTTATATGATTACATAGTTGCTACAACGTTCTATTAATCCCTTCAAATC
AATTATCTTCAAACAAGTTGCTG

[1] PCRs 1-12 indicate the relative primer positions as shown in Fig. 1a, Supplementary Fig. S2a, and Supplementary Fig. S5a. CT: carboxy-terminus; NTD: amino-terminal domain; HR: homology region; recod: recodonized sequence; F: forward primer; R: reverse primer. Oligonucleotides were purchased from Merck or IDT. DNA synthesis was performed by Azenta Life Sciences.

[2] Annealing bases are shown in upper case, overhangs in lower case.

[3] Expected PCR fragments for wild-type (WT) and mutant parasites.

[4] Complementary oligonucleotides were annealed, phosphorylated and cloned into the pDC2-Cas9-hDHFRyFCU plasmid using BbsI for the 3D7-based mutant. Alternatively, annealed oligonucleotides were inserted into AflII-digested pAIO-3 plasmid using In-Fusion cloning for the NF54-based mutant.

[5] Color code: HR start and stop codons intron *LoxP* sites recodonized HDP linker mNG smHA HSP86 3'UTR HRPII 3'UTR yDHODH calmodulin 5'UTR

Supplementary Table S2. **Transgenic parasite strains used in this study.**

Mutant	Parental strain[1]	Description[2]
B11	3D7	Constitutive expression of DiCre recombinase
hdp-mNG:loxP	B11	Conditional deletion of mNG-tagged HDP by DiCre
hdp-mNG:loxP + mito-mCh + ydhodh	*hdp-mNG:loxP*	Episomal expression of mitochondrial marker in HDP-mNG DiCre line
hdp-mNG:loxP + apico-mCh + bsd	*hdp-mNG:loxP*	Episomal expression of apicoplast marker in HDP-mNG DiCre line
hdp-mCh + ydhodh	3D7	Episomal expression of mCh-tagged HDP
ntd-mCh + ydhodh	3D7	Episomal expression of mCh-tagged HDP N-terminal domain
hdp-mNG:loxP + ydhodh	*hdp-mNG:loxP*	Phenotypic rescue of HDP-mNG DiCre line by yDHODH
hdp-mNG:loxP + cox5b-mCh	*hdp-mNG:loxP*	Endogenous mCh-tagging of Cox5B in HDP-mNG DiCre line
hdp-mNG:loxP + ndufa4-mCh	*hdp-mNG:loxP*	Endogenous mCh-tagging of NDUFA4 in HDP-mNG DiCre line
hdp-mNG:loxP + cox5b-mCh + ydhodh	*hdp-mNG:loxP + cox5b-mCh*	Phenotypic rescue of HDP-mNG DiCre line expressing mCh-tagged Cox5B by yDHODH
hdp-mNG:loxP + ndufa4-mCh + ydhodh	*hdp-mNG:loxP + ndufa4-mCh*	Phenotypic rescue of HDP-mNG DiCre line expressing mCh-tagged Cox5B by yDHODH
NF54$^{\text{attB-DiCre}}$	NF54^{attB}	Constitutive expression of DiCre recombinase
hdp-F91L-smHA:loxP + ydhodh	NF54$^{\text{attB-DiCre}}$	Conditional deletion of smHA-tagged HDP F91L by DiCre and rescue by yDHODH

[1] Recipient strains used as genetic background for parasite transfection.

[2] DiCre, dimerizable Cre recombinase; mCh, mCherry; mNG, mNeonGreen; yDHODH, yeast dihydroorotate dehydrogenase.

UNCROPPED GELS AND BLOTS FROM SUPPLEMENTARY FIGURES

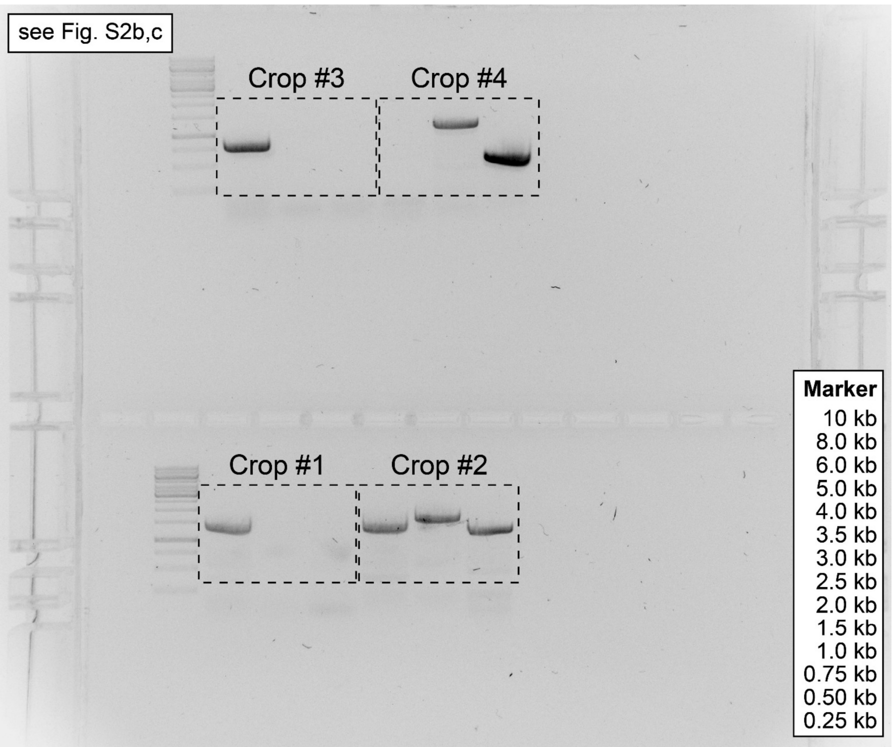

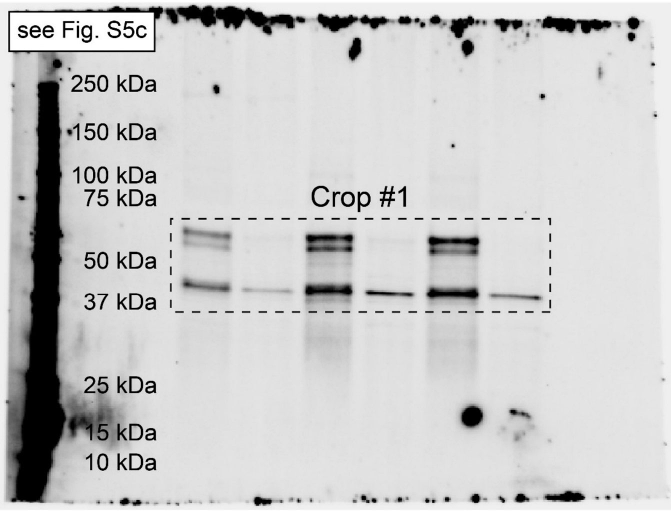

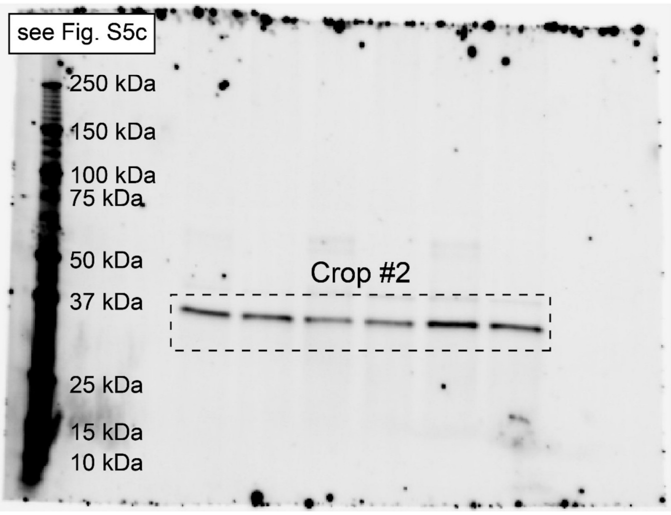

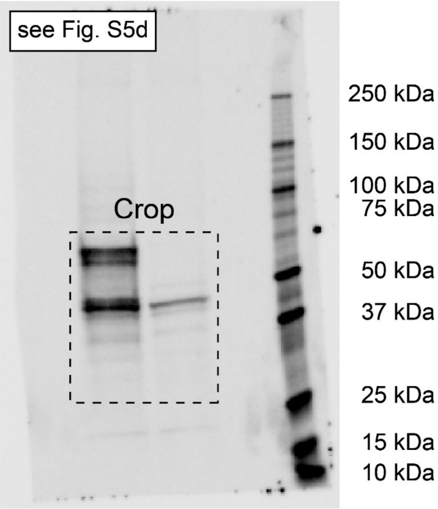

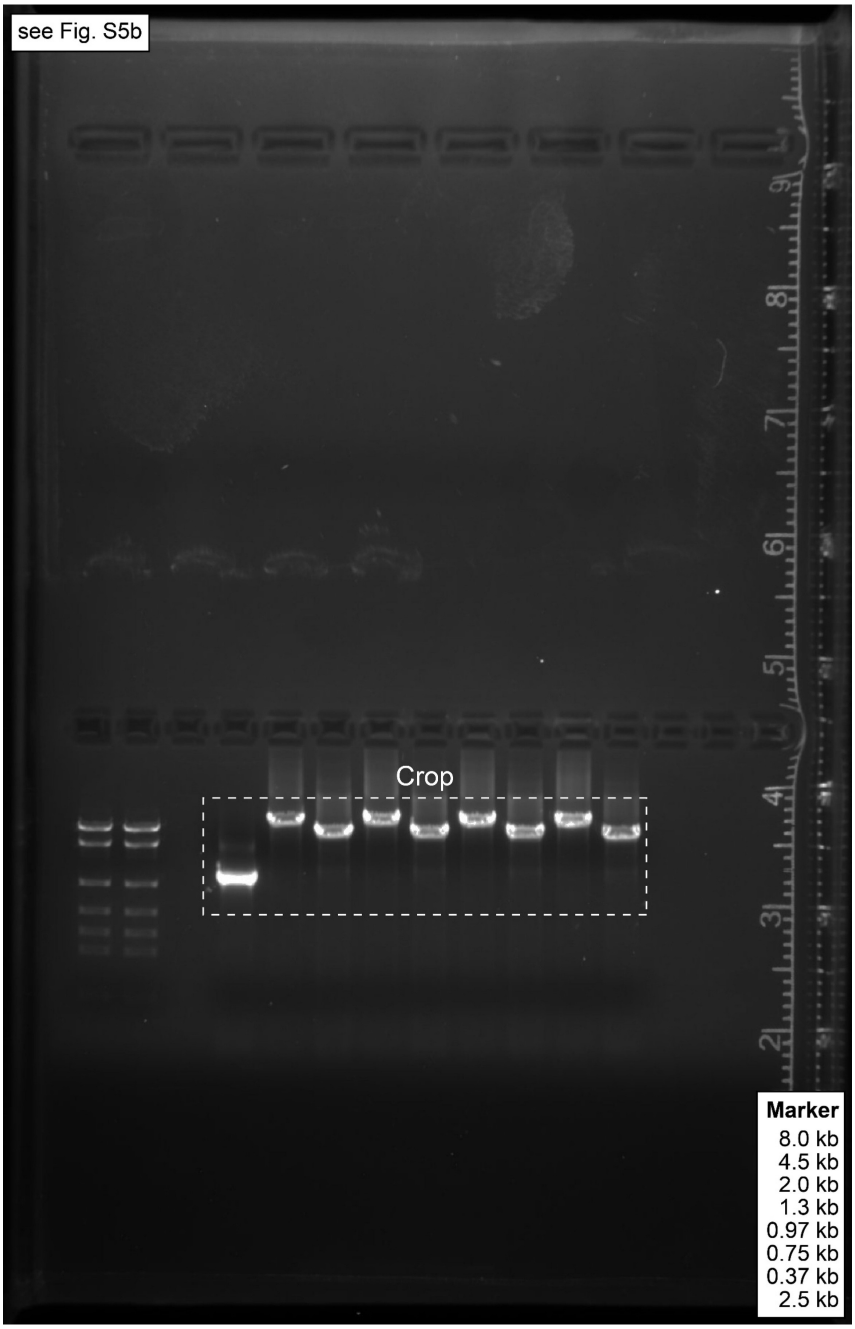

SUPPLEMENTARY REFERENCES

1. Birnbaum, J. *et al.* A genetic system to study *Plasmodium falciparum* protein function. *Nat Methods* **14**, 450-456 (2017).
2. Wang, C. *et al.* Apicomplexan mitoribosome from highly fragmented rRNAs to a functional machine. *Nat Commun* **15**, 10689 (2024).
3. Underhaug, J. *et al.* Mutation in transforming growth factor beta induced protein associated with granular corneal dystrophy type 1 reduces the proteolytic susceptibility through local structural stabilization. *Biochim Biophys Acta* **1834**, 2812-2822 (2013).
4. Lemoine, F. *et al.* NGPhylogeny.fr: new generation phylogenetic services for non-specialists. *Nucleic Acids Res* **47**, W260-W265 (2019).
5. Harb, O.S., McDowell, M.A. & Roos, D.S. VEuPathDB resources: a platform for free online data exploration, integration, and analysis. *Methods Mol Biol* **2802**, 573-586 (2024).
