# Supplementary material for: The Plasmodium heme detoxification protein functions in mitochondrial protein synthesis
Source: Nat Commun. 2026 Aug 7;17:8002. doi: 10.1038/s41467-026-76511-6 (PMC13451264; doi:10.1038/s41467-026-76511-6)
Supplement: Supplementary file 2 [file 41467_2026_76511_MOESM2_ESM.pdf]

<u>Description of Additional Supplementary Files</u>

Title: Supplementary Data 1

Description: Quantitative proteomic analysis of respiratory chain complexes and mitoribosomal subunits in *hdp-mNG:loxP + ydhodh* parasites.
