# Supplementary material for: The Plasmodium heme detoxification protein functions in mitochondrial protein synthesis
Source: Nat Commun. 2026 Aug 7;17:8002. doi: 10.1038/s41467-026-76511-6 (PMC13451264; doi:10.1038/s41467-026-76511-6)
Supplement: Supplementary file 3 [file 41467_2026_76511_MOESM4_ESM.pdf]

nature portfolio

Corresponding author(s): Joachim Michael Matz

Last updated by author(s): 07.05.2026

Reporting Summary

Nature Portfolio wishes to improve the reproducibility of the work that we publish. This form provides structure for consistency and transparency in reporting. For further information on Nature Portfolio policies, see our Editorial Policies and the Editorial Policy Checklist.

Statistics

For all statistical analyses, confirm that the following items are present in the figure legend, table legend, main text, or Methods section.

n/a	Confirmed	
☐	☒	The exact sample size (*n*) for each experimental group/condition, given as a discrete number and unit of measurement
☒	☐	A statement on whether measurements were taken from distinct samples or whether the same sample was measured repeatedly
☐	☒	The statistical test(s) used AND whether they are one- or two-sided<br>*Only common tests should be described solely by name; describe more complex techniques in the Methods section.*
☒	☐	A description of all covariates tested
☐	☒	A description of any assumptions or corrections, such as tests of normality and adjustment for multiple comparisons
☐	☒	A full description of the statistical parameters including central tendency (e.g. means) or other basic estimates (e.g. regression coefficient) AND variation (e.g. standard deviation) or associated estimates of uncertainty (e.g. confidence intervals)
☐	☒	For null hypothesis testing, the test statistic (e.g. *F*, *t*, *r*) with confidence intervals, effect sizes, degrees of freedom and *P* value noted<br>*Give P values as exact values whenever suitable.*
☒	☐	For Bayesian analysis, information on the choice of priors and Markov chain Monte Carlo settings
☒	☐	For hierarchical and complex designs, identification of the appropriate level for tests and full reporting of outcomes
☒	☐	Estimates of effect sizes (e.g. Cohen's *d*, Pearson's *r*), indicating how they were calculated

Our web collection on statistics for biologists contains articles on many of the points above.

Software and code

Policy information about availability of computer code

Data collection	AlphaFold 3, Agilent NovoExpress (version 1.6.1), BD FACSDiva Software (version 8), Leica Application Suite X (version 3.7.6.25997), AxioVision (version 4.8)
Data analysis	Graphpad Prism (version 10.4.1), DIA-NN (version 2.2.0), FIJI (version 2.16.0/1.54p), PyMOL (version 2.5.7), ChimeraX (version 1.10)

For manuscripts utilizing custom algorithms or software that are central to the research but not yet described in published literature, software must be made available to editors and reviewers. We strongly encourage code deposition in a community repository (e.g. GitHub). See the Nature Portfolio guidelines for submitting code & software for further information.

Data

Policy information about availability of data

All manuscripts must include a data availability statement. This statement should provide the following information, where applicable:

- Accession codes, unique identifiers, or web links for publicly available datasets
- A description of any restrictions on data availability
- For clinical datasets or third party data, please ensure that the statement adheres to our policy

The mass spectrometry data generated in this study have been deposited to the ProteomeXchange Consortium via the jPOST partner repository under accession codes PXD073350 [https://proteomecentral.proteomexchange.org/ui?pxid=PXD073350] and JPST004216 [https://repository.jpostdb.org/entry/JPST004216]. Whole-genome sequencing data are available in the NCBI Sequence Read Archive database under accession code PRJNA1416976 [https://www.ncbi.nlm.nih.gov/

bioproject/PRJNA1416976/]. All other data supporting the findings of this study are included in the article and its supplementary materials. Source data are provided with this paper. DNA constructs and transgenic parasite lines used in this study are available upon reasonable request.

Research involving human participants, their data, or biological material

Policy information about studies with human participants or human data. See also policy information about sex, gender (identity/presentation), and sexual orientation and race, ethnicity and racism.

Reporting on sex and gender	P. falciparum blood-stage parasites were cultured in deidentified human erythrocytes obtained from the Universitätsklinikum Hamburg-Eppendorf. Data on sex and gender of the donors is not available to us, but the parasites develop equally well in erythrocytes from male and female donors.
Reporting on race, ethnicity, or other socially relevant groupings	Blood donations have been deidentified by the Universitätsklinikum Hamburg-Eppendorf (see above)
Population characteristics	Blood donations have been deidentified by the Universitätsklinikum Hamburg-Eppendorf (see above)
Recruitment	Blood donations have been deidentified by the Universitätsklinikum Hamburg-Eppendorf (see above)
Ethics oversight	Not required

Note that full information on the approval of the study protocol must also be provided in the manuscript.

Field-specific reporting

Please select the one below that is the best fit for your research. If you are not sure, read the appropriate sections before making your selection.

☒ Life sciences ☐ Behavioural & social sciences ☐ Ecological, evolutionary & environmental sciences

For a reference copy of the document with all sections, see nature.com/documents/nr-reporting-summary-flat.pdf

Life sciences study design

All studies must disclose on these points even when the disclosure is negative.

Sample size	The sample size was determined a posteriori based on the observed variability. For quantitaitve assessments, at least three biological replicates were included.
Data exclusions	No data was excluded.
Replication	All attempts at replication were successful.
Randomization	Not applicable.
Blinding	Blinding was not performed, as all outcome measures were quantitative, objective, and not subject to subjective interpretation during data acquisition or analysis.

Reporting for specific materials, systems and methods

We require information from authors about some types of materials, experimental systems and methods used in many studies. Here, indicate whether each material, system or method listed is relevant to your study. If you are not sure if a list item applies to your research, read the appropriate section before selecting a response.

Materials & experimental systems

n/a	Involved in the study	
☐	☒	Antibodies
☐	☒	Eukaryotic cell lines
☒	☐	Palaeontology and archaeology
☒	☐	Animals and other organisms
☒	☐	Clinical data
☒	☐	Dual use research of concern
☒	☐	Plants

Methods

n/a	Involved in the study	
☒	☐	ChIP-seq
☐	☒	Flow cytometry
☒	☐	MRI-based neuroimaging

Antibodies

Antibodies used	α-BiP (rabbit, 1:2,000), E. Knuepfer et al., RON12, a novel Plasmodium-specific rhoptry neck protein important for parasite proliferation. Cell Microbiol 16, 657-672 (2014);<br>α-mNG (ChromoTek, mouse, 1:1,000, 32F6, Catalog-No.:17353263);<br>α-RFP (ChromoTek, rat, 1:1,000, 5F8, Catalog-No.: 5f8);<br>α-HA (Merck, rabbit, 1:1,000, Catalog-No.: H6908);<br>α-aldolase (abcam, rabbit, 1:1,000, Catalog-No.: ab38905)
Validation	α-BiP: undisclosed<br>α-mNG: Western blot, IFA<br>α-RFP: IFA<br>α-HA: IFA, Western blot, IP<br>α-aldolase: Western blot, ELISA

Eukaryotic cell lines

Policy information about cell lines and Sex and Gender in Research

Cell line source(s)	Only P. falciparum (non-vertebrate) cell lines were used.
Authentication	Not applicable.
Mycoplasma contamination	P. falciparum cell lines were not tested for Mycoplasma.
Commonly misidentified lines<br>(See ICLAC register)	Not applicable.

Plants

Seed stocks	Not applicable.
Novel plant genotypes	Not applicable.
Authentication	Not applicable.

Flow Cytometry

Plots

Confirm that:

- [ ] The axis labels state the marker and fluorochrome used (e.g. CD4-FITC).
- [ ] The axis scales are clearly visible. Include numbers along axes only for bottom left plot of group (a 'group' is an analysis of identical markers).
- [ ] All plots are contour plots with outliers or pseudocolor plots.
- [ ] A numerical value for number of cells or percentage (with statistics) is provided.

Methodology

Sample preparation	Parasites were stained for 40 minutes at 37°C in medium containing SYBR Green nuclear dye (1:10,000; Thermo Fisher Scientific), followed by a 40-minute de-staining period.
Instrument	NovoCyte 3000VYB flow cytometer equipped with a NovoSampler Pro (Agilent).
Software	NovoExpress Software (Agilent)
Cell population abundance	Not applicable, as parasites were not sorted.

Gating strategy

After eliminating debris, singlet cells were gated based in forward and sidescatter. From that population, the proportion of SYBR-Green-positive cells was determined.

☐ Tick this box to confirm that a figure exemplifying the gating strategy is provided in the Supplementary Information.
