# Supplementary material for: The Plasmodium heme detoxification protein functions in mitochondrial protein synthesis
Source: Nat Commun. 2026 Aug 7;17:8002. doi: 10.1038/s41467-026-76511-6 (PMC13451264; doi:10.1038/s41467-026-76511-6)
Supplement: Supplementary file 4 [file 41467_2026_76511_MOESM5_ESM.pdf]

nature portfolio

Peer Review File

The *Plasmodium* heme detoxification protein functions in mitochondrial protein synthesis

Corresponding Author: Dr Joachim Matz

This file contains all reviewer reports in order by version, followed by all author rebuttals in order by version.

Version 0:

Reviewer comments:

Reviewer #1

(Remarks to the Author)
This manuscript by Sarrazin, Matz and coworkers investigates the location and role of the hemozoin detoxification protein (HDP) within the malaria parasite, previously thought to be involved in hemozoin formation. The authors used a systematic approach to define the role of HDP, beginning with its localization within P. falciparum in vitro cultures. They demonstrate that HDP localizes to the mitochondrion rather than the digestive vacuole as previously proposed. They next show that HDP is required for erythrocytic parasite survival in the second replication cycle, coinciding with a loss of hemozoin formation at a similar time point. Given that a comparable reduction in hemozoin content is tolerated in an unrelated mutant, this suggests that HDP is essential for parasite survival through a function distinct from heme biomineralization. These findings, together with heme fractionation and analysis of hemozoin quantity and morphology, provide strong evidence supporting the conclusion that HDP does not function directly in hemozoin formation.

Given the localization of HDP in the mitochondrion, the authors investigate its role in the mitochondrial electron transport chain and show that HDP is required to maintain mitochondrial membrane potential. They further demonstrate that bypassing the electron transport chain can rescue HDP-deficient parasites and that rescued parasites exhibit significantly reduced respiration. Additionally, the authors present evidence that HDP plays a role in mitochondrial protein biosynthesis and propose that it might be an integral component of the mitoribosomal large subunit.

Overall, this research provides strong evidence for an essential role of HDP in mitochondrial protein biosynthesis, rather than hemozoin formation. This work will have a significant and defining impact on the field and be of interest to a wide range of readers. The data is of high quality and robust, and the manuscript is logically and clearly written. We recommend this work for publication in Nature Communications after some minor edits as per the below comments.

Comments:
Figures 1B and 2A-B (and all other gels): For Nature Comms articles, any PCR or gel electrophoresis image must include a molecular weight marker (ladder) to be considered scientifically valid and reproducible.Figure 1E/F: Please define the abbreviations mCh, Apico-mCh, and Mito-mCh in the figure legend.

Pg. 5, line 146 and 151-152: “We overexpressed HDP fused to mCherry (mCh)” and “we further modified the hdp-mNG:loxP parasites to episomally express minimal mCh-based reporters for both these organelles.” seems to be glossed over without giving experimental details or evidence or a reference for these cell lines. Could this please be added to (or referenced from) the supplementary information.

Figure 2H: Please include RAP/DMSO key on the graph for complete clarity and consistency with other graphs.

Pg. 6, lines 180-181 and Figure 2H/I: The text states that “samples were then collected every two hours, and parasite size was quantified microscopically in methanol-fixed blood smears.” However, the graphs show data points at hourly intervals post-invasion. This inconsistency should be clarified.

Figure 2D/H/I and Figure 3A: The time representation for cycle 1 and cycle 2 is inconsistent across figures. For example, Figures 2H/I could present time points as continuous hours from the start of cycle 1 to allow direct comparison with the microscopy images in Figure 2D. Alternatively, the representation of time points in Figure 2D could be adjusted to match the Figures 2H/I and 3A.

Pg. 6 line 219 and 221-222 and Figures 3E-F: Could more details and evidence please be provided for “the hdp-mNG:loxP + mito-mCh + ydhodh mutant” and “hdp-mNG:loxP + apico-mCh + bsd parasites” via referencing and/or adding to the supplementary info.

Pg. 7 lines 234-245 and Figure 3H: The addition of sodium azide is shown in Figure 3H but is not mentioned in the text. Please specify the purpose of adding sodium azide and whether the reduction in oxygen consumption rate in DMSO-treated parasites was expected.

Pg 7 lines 246-247 and Figure 3J: Could more details and evidence please be provided for "we endogenously tagged the subunits NDUFA4 and Cox5B with mCh in a yDHODH-rescued hdp-mNG:loxP background" via referencing and/or adding to the supplementary info.

Figure 5F-H and pg. 15 Heme fractionation assay: The heme fractionation assay requires additional methodological detail to detail the deviations from the literature protocol. The authors should specify the culture volume, percentage parasitemia, and hematocrit used in this assay. We also question the use of 100 nM chloroquine (CQ) concentration in this assay, as this is approximately 8× the CQ IC50 in PfNF54, whereas we generally test up to 3× IC50. Furthermore, the incubation time of 16 hours is a lot shorter than what is generally used (28-32 hours). Providing further mechanistic details and justification would help clarify these choices.

Figure 5F-H: The figure legend should specify that relative hemozoin, hemoglobin, and heme refer to the absolute amounts of each species per parasite (as opposed to % amounts), normalized to the absolute amount in the DMSO-treated, no drug control. The statistical analysis used should also be included in the legend.

Pg. 9, line 323-325 and pg. 33 Supplementary Figure S3F: The authors state that RAP treatment sensitizes the hdp-smHA:loxP + ydhodh NF54 line to proguanil. However, the data in Supplementary Figure S3F appear to show the opposite trend. The DMSO and RAP curves may have been incorrectly labelled.

Pg. 8, line 320: Change "haemagglutinin" to "hemagglutinin" to maintain consistency with American English throughout the manuscript.

Pg. 10 Line 362, 363, and 385: Define the abbreviations GFP, Co-IP, HRP upon their first appearance.

Reviewer #2

(Remarks to the Author)
I co-reviewed this manuscript with one of the reviewers who provided the listed reports. This is part of the Nature Communications initiative to facilitate training in peer review and to provide appropriate recognition for Early Career Researchers who co-review manuscripts.

Reviewer #3

(Remarks to the Author)
Having previously studied aspects of the Plasmodium falciparum (Pf) digestive vacuole physiology, an important antimalarial drug target, L. Sarrazin, J. Matz and colleagues turn their attention in the studies reported in this manuscript to Pf HDP (heme detoxification protein), thought to be critical for heme biomineralization in the digestive vacuole. Using a series of transgenic Pf lines, in which HDP is tagged and/or subject to conditional knockout, the authors find that (tagged) HDP is primarily found in the parasite mitochondrion rather than the previously reported locations (parasite cytoplasm and digestive vacuole and RBC cytoplasm), and depletion of PfHDP did not affect hemozoin formation. HDP was, however, found to be critical for maintenance of mitochondrial electron transport and membrane polarization. Deletion of the hdp gene was lethal but lethality could be overcome by the transgenic expression of yDHODH (yeast dihydroorotate dehydrogenase), which provides an alternate path to pyrimidine biosynthesis not linked to ubiquinone and the electron transport chain (ETC). Δhdp (ydhodh+) lines were hypersensitive to proguanil, the synergistic partner drug to the ETC inhibitor atovaquone in the antimalarial Malarone. Loss of atovaquone-sensitive oxygen consumption was also observed in Δhdp (ydhodh+) parasites. Structural comparisons using AlphaFold structural predictions and experimental structures, where available, confirmed the likely ortholog of PfHDP in Toxoplasma, TGME49_285272, which is a mitoribosomal protein (mL135) located at the LSU-SSU interface. Indeed, loss of hdp correlated with depletion of many ETC complex subunits and mitoribosomal proteins, as observed in proteomics experiments.
In general, the manuscript was well-written. The data was well presented and substantive and supported by appropriate statistical tests when appropriate. This work should be of interest to parasitologists, especially malariologists, tropical medicine and drug development scientists.

There are a few points I think should be clarified.

One area concerns the construction and validation of the transgenic Pf lines. The sequences of two principal repair constructs are provided in the supplementary materials. First, in the hdp-smHA:loxP + ydhodh construct, there is a nonsynonymous change in the coding sequence resulting in a Phe to Leu substitution at aa position 91. Is there a rationale for this?
MKNRFYYNLIIKRLYTRSGGLRKPQKVTNDPESINRKVYWCFEHKPVKRTIINLIYSHNELKIFSNLLNHPTVGSSLIHELSLDGPYTAFL…

Nanopore/whole genome sequencing is part of the validation of the resulting lines, and some nanopore reads are provided in the supplementary materials. The sequence from the RAP-treated deletion line was puzzling; self-BLAST dot-plot indicated the presence of repeated sequences at the 5' end. I mapped the elements present and observed ~2100 bp at the 5' end that consists of an (imperfect) inverted repeat, which includes 2 loxP elements (a normal CRE-lox deletion would leave only a single loxP). The provided sequences do not extend into the surrounding genomic DNA, so we cannot be sure that this apparent rearrangement has not caused changes to the surrounding loci.
This question should be addressable in the whole genome sequence alluded to in the manuscript, but this data doesn't seem to be easily analyzed by the average reviewer/reader on their PC/laptop; hopefully there were no changes that would affect the results, and in that case I just suggest that additional accessible sequence data be provided that covers the repair-genomic DNA junctions and immediately surrounding loci.

In the methods describing the generation of the 3D7-based hdp-mNG:loxP line, it describes "…the mNG tag was cloned in-frame using HpaI and EcoRV restriction sites." [manuscript lines 458-458], but we are not informed what plasmid contains these sites; it should be described here, along with the plasmid used to express Cas9 and the sgRNAs (to allow for reproduction of the work). Also the method of excising the "linearized repair template" should be described; judging by Fig. 1A, it looks like the repair template is cut out of its host plasmid without carrying along any of the plasmid in contrast to another commonly used method, in which the cut site(s) are placed between the 3' HR and 5' HR so that almost all of the plasmid, including a selectable marker, are included in the linearized construct and incorporated in the genome following repair.

In Figure 1A diagram of the hdp-mNG:loxP line generation process, the position of primer P5-R is misrepresented. P4-R should be about where P5-R is shown (well downstream of the 3'HR), and P5-R should be close to the middle of the 3' HR. The position of the primers is relevant as it allows the reader to see which PCR reactions verify the insertion-genomic DNA junctions and which report elements internal to the repair construct inserts. Similarly, in Fig S3A, primer P6-R should be shown downstream of the 3' UTR. Additionally, The P3-R and P4-F primer binding sequences are located in the loxP elements, ie., they each occur 2X in the repair templates. Is there a reason for this, and one wonders why only the shorter of the possible PCR products appears in each case? If the PCR conditions were adjusted to favor short products, this should be described in the Methods section.

Other minor suggestions/questions follow:

Lines 93-95: The question of reaction volume and reaction essentiality are not necessarily directly linked. Are there actual measurements on this question? In the absence of metabolic flux measurements of the relative reaction mass processed by each quinone-dependent dehydrogenase in wild type blood stage parasites during normal growth, one cannot say whether one dehydrogenase donates significantly more electrons to the ETC than each or all of the others. If this is the case, then statements about minor and main electron donating enzyme reactions should be removed.

Lines 121-122: Recently transposon screens of P. knowlesi were reported. Elsworth et al (2025) had particularly dense coverage and also found that the HDP ortholog was likely essential in Pkn. If desired, this could be cited here to increase the range of spp. in which HDP appears to be essential. [Elsworth B, Ye S, Dass S, Tennessen JA, Sultana Q, Thommen BT, Paul AS, Kanjee U, Grüring C, Ferreira MU, Gubbels MJ, Zarringhalam K, Duraisingh The essential genome of Plasmodium knowlesi reveals determinants of antimalarial susceptibility. MT.Science. 2025;387(6734):eadq6241. doi: 10.1126/science.adq6241.]

Line 133: "…conflicting localizations for HDP," Under the previous hypothesis for trafficking of HDP to the DV these were thought to be sequential steps of the pathway, as the authors here discuss elsewhere in the manuscript. I suggest using phrasing like "multiple localizations".

Lines 135-136 and Fig. 1: Note that the mNG tag is larger than HDP (the smHA tag is even larger). Large tags have been known to sometimes cause mislocalization; this caveat should be mentioned (and discounted if appropriate). Since the localization of HDP shown here differs from that reported previously, possible reasons for the differences should be discussed somewhere in this manuscript.

In Fig 2 H& I, the meaning of the data bar colors in the graphs should be described in the caption.
Fig 3J. The authors should probably quantitate the relative band intensities using software such as ImageJ or Fiji.

Fig. 4A. The shading of the numbers in the alignment is faint, making it hard to perceive colors mentioned in the caption. Perhaps these could be darker.

Lines `265-278 and Fig. 4 examine the sequence and, especially, structural similarities of Pf HDP to Fas1 domain proteins in Toxoplasma gondii, concluding that TGME49_285272 (mL135 in Shikha et al mitoribosome structure) is the likely ortholog of Pf HDP. If Pf HDP is an important mitoribosomal protein conserved in Toxoplasma and Plasmodium, which are not the closest evolutionary "cousins", one would expect orthologues in other Apicomplexan species with mitochondria and perhaps other in related groups. It would be of interest, then, to determine the extent of such putative orthologues in Apicomplexa and related phyla. A few quick BLAST searches found such apparent orthologues in Theileria, Babesia, Chomera and Vitrella spp., for example.
TA11080 hypothetical protein, conserved (with C-terminal Fas1 domain)
BBOV_III009730 heme detoxification protein (fasciclin I)
Cvel_24500 Heme ligase, putative (has C-terminal Fas1 domain)
Vbra_10965 Heme ligase, putative (has C-terminal Fas1 domain)

Fig. 4E. pLDDT should be defined/described.

In line 463, "In-Fusion cloning" should be described or referenced.

Reviewer #4

(Remarks to the Author)
The paper titled "Redefining the role of the Plasmodium heme detoxification protein: From hemozoin formation to mitochondrial protein synthesis," by Sarrazin et al., aims to address an interesting and long-standing question in malaria biology: the function and localization of the heme detoxification protein (HDP). The paper is well-written, clearly structured, and the experiments were performed rigorously, offering an interesting new perspective on HDP biology that is sure to interest scientists working on various aspects of hemoglobin metabolism, hemozoin biosynthesis, and parasite organellar biology.
What is interesting about this paper is the attempt to revisit the traditional model of hemozoin biosynthesis, in which HDP is known to play a role. The section on HDP localization, however, could benefit from integrating the earlier work on this topic to define current thinking on heme detoxification in Plasmodium falciparum.

The original work by Jani et al. (2008) identified HDP as a heme-binding protein that promotes β-hematin formation in vitro. In that study, the authors proposed an unusual trafficking pathway in which HDP is exported to the cytosol of the infected erythrocyte and subsequently re-internalized during hemoglobin endocytosis, eventually accumulating in the digestive vacuole. Based on these observations, HDP was suggested to participate directly in detoxifying free heme released during hemoglobin degradation.
Subsequent work has refined the mechanistic understanding of hemozoin formation. In particular, Matz et al. (2020) demonstrated that the digestive-vacuole protein PV5, a lipocalin-like protein, plays a regulatory role in hemozoin crystal growth and organization. Rather than directly catalyzing heme crystallization, PV5 appears to guide the ordered assembly of hemozoin within the digestive vacuole. These findings suggested that hemozoin biomineralization may be controlled by specialized vacuolar proteins and structural regulators, rather than relying exclusively on HDP-mediated catalysis.
Major points:
The detection of HDP in the erythrocytic cytosol following streptolysin-O-induced permeabilization should be interpreted with caution. This treatment can disrupt parasite integrity and release intracellular organelles, potentially exposing parasite proteins outside their native compartments.
At the same time, the absence of a strong HDP signal in the digestive vacuole when the protein is fused to mNeonGreen does not necessarily exclude a transient localization in this compartment. Fluorescent proteins often lose signal in acidic environments, such as the digestive vacuole, complicating the interpretation of fluorescence-based localization experiments. Therefore, it remains possible that HDP transiently traffics through the digestive vacuole or participates in a shuttle-type pathway linked to hemoglobin processing.
To further clarify this issue, complementary biochemical approaches could be extremely informative. For example, isolating parasite mitochondria (https://doi.org/10.1038/nature05572) and digestive vacuoles (https://doi.org/10.1016/S0006-2952(98)00140-3), followed by immunoblotting with anti-HDP antibodies, could provide direct evidence of HDP enrichment in specific organellar fractions through the parasite cell cycle. Such experiments would help clarify the current interpretation in relation to previous studies and strengthen the conclusions regarding HDP localization.
It would also be valuable for the authors to review the following work on mitochondrial biology in Plasmodium falciparum. Scarpelli et al. (2018) showed that mitochondrial dynamics in the parasite are regulated through fission-related proteins, highlighting the importance of mitochondrial homeostasis for parasite survival and development. More recently, Sparkes, Penny C., et al. (2024) examined mitochondrial function during gametocytogenesis and demonstrated that mitochondrial activity and ATP production are important for parasite differentiation. Although this study focuses on sexual-stage development, it shows the mitochondrion's central role in coordinating key cellular processes in the parasite. These studies collectively support the view that the parasite mitochondrion functions within a broader cellular network rather than as an isolated organelle. In this light, discussing this literature could strengthen the possibility that HDP localization to mitochondria reflects a previously unrecognized connection between mitochondrial function and pathways related to hemoglobin metabolism and heme detoxification.
Overall, this manuscript presents a stimulating reinterpretation of HDP function and opens new perspectives on the relationship between hemoglobin metabolism, heme detoxification, and parasite organellar biology. With a more comprehensive discussion of earlier literature and additional consideration of alternative interpretations of HDP localization, the study will make an important contribution to malaria parasite cell biology and to the ongoing debate over the molecular mechanisms underlying hemozoin formation.

Version 1:

Reviewer comments:

Reviewer #1

(Remarks to the Author)
The authors have addressed the concerns raised in the initial review. They have provided additional methodological detail and justified their drug treatment regimen for the heme fractionation assay. They have also corrected errors identified in the figures, included molecular weight markers for all gels, added explanations where requested, and ensured that all abbreviations are now properly defined upon first use.
I would prefer that the word "formation" is kept in the title to read:
Redefining the role of the Plasmodium heme detoxification protein: From hemozoin formation to mitochondrial protein synthesis
It doesn't really make sense without the word "formation".
Otherwise, I support acceptance of this manuscript for publication in Nature Communications.

Reviewer #2

(Remarks to the Author)
I co-reviewed this manuscript with one of the reviewers who provided the listed reports. This is part of the Nature Communications initiative to facilitate training in peer review and to provide appropriate recognition for Early Career Researchers who co-review manuscripts.

Reviewer #4

(Remarks to the Author)
The authors have performed the additional experiments requested and have satisfactorily addressed all of my comments and queries. I am therefore satisfied with the revisions made and believe that the manuscript has been significantly improved. In its current form, I support its publication.

Open Access This Peer Review File is licensed under a Creative Commons Attribution 4.0 International License, which permits use, sharing, adaptation, distribution and reproduction in any medium or format, as long as you give appropriate credit to the original author(s) and the source, provide a link to the Creative Commons license, and indicate if changes were made.
In cases where reviewers are anonymous, credit should be given to 'Anonymous Referee' and the source.
The images or other third party material in this Peer Review File are included in the article's Creative Commons license, unless indicated otherwise in a credit line to the material. If material is not included in the article's Creative Commons license and your intended use is not permitted by statutory regulation or exceeds the permitted use, you will need to obtain permission directly from the copyright holder.
To view a copy of this license, visit https://creativecommons.org/licenses/by/4.0/

Response to reviewers

Reviewer #1

This manuscript by Sarrazin, Matz and coworkers investigates the location and role of the heme detoxification protein (HDP) within the malaria parasite, previously thought to be involved in hemozoin formation. The authors used a systematic approach to define the role of HDP, beginning with its localization within *P. falciparum in vitro* cultures. They demonstrate that HDP localizes to the mitochondrion rather than the digestive vacuole as previously proposed. They next show that HDP is required for erythrocytic parasite survival in the second replication cycle, coinciding with a loss of hemozoin formation at a similar time point. Given that a comparable reduction in hemozoin content is tolerated in an unrelated mutant, this suggests that HDP is essential for parasite survival through a function distinct from heme biomineralization. These findings, together with heme fractionation and analysis of hemozoin quantity and morphology, provide strong evidence supporting the conclusion that HDP does not function directly in hemozoin formation. Given the localization of HDP in the mitochondrion, the authors investigate its role in the mitochondrial electron transport chain and show that HDP is required to maintain mitochondrial membrane potential. They further demonstrate that bypassing the electron transport chain can rescue HDP-deficient parasites and that rescued parasites exhibit significantly reduced respiration. Additionally, the authors present evidence that HDP plays a role in mitochondrial protein biosynthesis and propose that it might be an integral component of the mitoribosomal large subunit.

Overall, this research provides strong evidence for an essential role of HDP in mitochondrial protein biosynthesis, rather than hemozoin formation. This work will have a significant and defining impact on the field and be of interest to a wide range of readers. The data is of high quality and robust, and the manuscript is logically and clearly written. We recommend this work for publication in *Nature Communications* after some minor edits as per the below comments.

> We thank Reviewers #1 and #2 for their careful evaluation and constructive feedback.

Figures 1B and 2A-B (and all other gels): For *Nature Comms* articles, any PCR or gel electrophoresis image must include a molecular weight marker (ladder) to be considered scientifically valid and reproducible.

> We now provide all gels and blots in uncropped form within the Source Data file (Supplementary Data S2), clearly showing the edges and markers.

Figure 1E/F: Please define the abbreviations mCh, Apico-mCh, and Mito-mCh in the figure legend.

> We have now included this information in the revised Figure legend (Lines 848-850):
>
> > *"mNG: mNeonGreen; mCh: mCherry; Apico-mCh: mCherry fused to the apicoplast-targeting sequence of acyl carrier protein; Mito-mCh: mCherry fused to the mitochondrial targeting sequence of citrate synthase."*

Pg. 5, line 146 and 151-152: "We overexpressed HDP fused to mCherry (mCh)" and "we further modified the *hdp-mNG:loxP* parasites to episomally express minimal mCh-based reporters for both these organelles." seems to be glossed over without giving experimental details or evidence or a reference for these cell lines. Could this please be added to (or referenced from) the supplementary information.

> In our initial manuscript, we had included references for the episomal Apico-mCh and Mito-mCh expression constructs in the Methods section (previously Lines 474-476):
>
> > *"Plasmids for episomal expression of the apicoplast (84) and mitochondrial (65) markers were maintained using 2 µg/mL blasticidin S (Thermo Fisher Scientific) or 0.9 µM DSM1, respectively."*

However, it might not have been clear enough that the mentioned organelle markers referred specifically to Apico-mCh and Mito-mCh. We have therefore rephrased the corresponding section for improved clarity and provided more information on the promoters, selection cassettes, and localization signals used, as outlined in the Supplementary Methods section:

> *"The Mito-mCh marker was expressed from the previously described episomal plasmid pNMD3:Mito-mCherry-DHODH[6], which carries a yDHODH expression cassette for selection with 0.9 µM DSM1. In this construct, mCh is fused to the leader sequence of citrate synthase and expressed under the control of the nonsense-mediated decay 3 (NMD3) promoter. Episomal constructs for expression of mCh-tagged HDP or the HDP NTD were generated by replacing the citrate synthase leader sequence with either the full-length HDP sequence or its first 48 residues, respectively, using the XhoI and KpnI restriction sites. For certain experiments, the Mito-mCh marker was excised using SnaBI and SmaI, allowing episomal yDHODH expression without co-expression of additional fluorescent markers. The Apico-mCh marker was expressed from the episomal plasmid pACP-mCherry_BSDcrt[7], which harbors a blasticidin S deaminase cassette for selection with 2 µg/mL blasticidin S (Thermo Fisher Scientific). In this construct, mCh is fused to the apicoplast targeting sequence of acyl carrier protein and expressed from the CRT promoter. Introduction of pNMD3:Mito-mCherry-DHODH and pACP-mCherry_BSDcrt into hdp-mNG:loxP parasites resulted in the hdp-mNG:loxP + mito-mCh + ydhodh and hdp-mNG:loxP + apico-mCh + bsd mutant lines"*

In addition, as mentioned above, we include a more detailed explanation of the markers in the corresponding Figure legend (Lines 848-850):

> *"Apico-mCh: mCherry fused to the apicoplast targeting sequence of acyl carrier protein; Mito-mCh: mCherry fused to the mitochondrial targeting sequence of citrate synthase."*

The emergence of drug-resistant parasites was considered sufficient evidence for successful introduction of the episomal plasmids, particularly because the parasites exhibited the expected fluorescent signals.

Figure 2H: Please include RAP/DMSO key on the graph for complete clarity and consistency with other graphs.

This has now been incorporated into the revised Figure.

Pg. 6, lines 180-181 and Figure 2H/I: The text states that "samples were then collected every two hours, and parasite size was quantified microscopically in methanol-fixed blood smears." However, the graphs show data points at hourly intervals post-invasion. This inconsistency should be clarified.

We thank the reviewer for pointing out this inconsistency. The samples were indeed collected hourly, and we have corrected the sentence accordingly (Lines 200-201):

> *"Samples were then collected every hour, and parasite size was quantified microscopically in methanol-fixed blood smears."*

Figure 2D/H/I and Figure 3A: The time representation for cycle 1 and cycle 2 is inconsistent across figures. For example, Figures 2H/I could present time points as continuous hours from the start of cycle 1 to allow direct comparison with the microscopy images in Figure 2D. Alternatively, the representation of time points in Figure 2D could be adjusted to match the Figures 2H/I and 3A.

We thank the reviewer for raising this point. This is not an error, but rather reflects differences in how the experiments were performed. For the Giemsa images, we started with synchronous ring-stage parasites and allowed them to develop through two full cycles. However, determining

the exact age of a parasite at 68 hours post-invasion is not straightforward. Assuming a fixed 48-hour cycle would place these parasites 20 hours into the second cycle, but in our lab, the cycle is typically shorter.

Observing that all relevant effects occur during cycle 2, we therefore implemented a second synchronization step at the end of the first cycle for all subsequent experiments, ensuring that they consistently started with tightly synchronized second-cycle ring stages. This change also accounts for the differences in labeling.

We already included a description of this approach in the Results section (Lines 198-200):

"To more precisely determine when developmental arrest occurs following HDP knockout, we treated ring-stage cultures with DMSO or RAP, allowed them to develop into mature schizonts, and used these schizonts to initiate a new, highly synchronous invasion."

To further ensure uniformity, we have now repeated the Giemsa smearing assay with this second synchronization step, allowing parasite age to be labeled consistently across all Figures. We also describe this procedure more explicitly in the Methods section of the revised manuscript (Lines 501-502):

"Parasites were resynchronized at the end of the first cycle to start experiments with second-cycle ring stages of precisely defined age."

Pg. 6 line 219 and 221-222 and Figures 3E-F: Could more details and evidence please be provided for "the *hdp-mNG:loxP* + *mito-mCh* + *ydhodh* mutant" and "*hdp-mNG:loxP* + *apico-mCh* + *bsd* parasites" via referencing and/or adding to the supplementary info.

As described above, this has been added to the Supplementary Methods section:

"*Introduction of pNMD3:Mito-mCherry-DHODH and pACP-mCherry_BSDcrt into hdp-mNG:loxP parasites resulted in the hdp-mNG:loxP + mito-mCh + ydhodh and hdp-mNG:loxP + apico-mCh + bsd mutant lines."*

Pg. 7 lines 234-245 and Figure 3H: The addition of sodium azide is shown in Figure 3H but is not mentioned in the text. Please specify the purpose of adding sodium azide and whether the reduction in oxygen consumption rate in DMSO-treated parasites was expected.

Sodium azide inhibits complex IV, thereby suppressing oxygen consumption. Accordingly, the decrease in OCR observed in DMSO-treated parasites is expected. Since HDP-deficient parasites exhibit minimal respiration to begin with, sodium azide had little to no effect on them. We have added a corresponding passage to the revised Results section (Lines 263-265):

"As expected, subsequent inhibition of complex IV with sodium azide reduced oxygen consumption in DMSO-treated controls, but did not further affect HDP-null parasites (Fig. 3h)."

Pg 7 lines 246-247 and Figure 3J: Could more details and evidence please be provided for "we endogenously tagged the subunits NDUFA4 and Cox5B with mCh in a yDHODH-rescued *hdp-mNG:loxP* background" via referencing and/or adding to the supplementary info.

In our previous submission, we provided details on vector construction and the overall strategy for tagging these proteins. We now additionally include a recombination schematic illustrating the genetic approach, together with genotyping PCRs and representative fluorescence images, as a new Supplementary Figure S2. The corresponding genotyping primers are listed in the revised Supplementary Table S1. The experimental approaches and validation strategies are

now described in more detail in the Supplementary Methods and the Results section of the revised manuscript (Lines 268-269):

"Endogenous tagging of Cox5B (PF3D7_0927800) and NDUFA4 (PF3D7_1439600) was achieved using the selection-linked integration system (Supplementary Fig. S2a)[8]. Tagging vectors were derived from the pSLI-PF3D7_1476700-mNeonGreen-glmS plasmid[9] and generated by first replacing mNG with mCh using the MluI and SalI sites, followed by cloning of the respective C-terminal region in frame with mCh using the NotI and MluI sites. After transfection, parasites were selected with WR99210 until resistant populations emerged and then treated with 0.4 mM neomycin (Merck) to enrich for integrants. Phenotypic rescue was achieved by subsequent transfection of the tagged lines with the episomal yDHODH-encoding plasmid. Correct genomic integration and mitochondrial mCh expression were validated by diagnostic PCR and live fluorescence microscopy, respectively (Supplementary Fig. S2b-e)"

"Correct genomic integration and expression of the tagged proteins were confirmed by diagnostic PCR and fluorescence microscopy, respectively (Supplementary Fig. S2b-e)."

Figure 5F-H and pg. 15 Heme fractionation assay: The heme fractionation assay requires additional methodological detail to detail the deviations from the literature protocol. The authors should specify the culture volume, percentage parasitemia, and hematocrit used in this assay. We also question the use of 100 nM chloroquine (CQ) concentration in this assay, as this is approximately 8× the CQ IC_{50} in PfNF54, whereas we generally test up to 3× IC_{50}. Furthermore, the incubation time of 16 hours is a lot shorter than what is generally used (28-32 hours). Providing further mechanistic details and justification would help clarify these choices.

We have now included the culture volume, hematocrit, and parasitemia used in the heme fractionation assays in the revised Methods section (Lines 606-608):

"For the heme fractionation assays, 2-mL cultures of hdp-F91L-smHA:loxP + ydhodh NF54 parasites at 4% hematocrit and 6-8% parasitemia were induced at the ring stage and incubated with or without 100 nM chloroquine for 16 hours, starting 56 hours post-induction."

All other minor deviations, including differences in drug concentrations, treatment times, and parasite quantification, were already noted.

Our drug treatment regimen differs somewhat from previous reports, as we used slightly higher chloroquine concentrations but for shorter treatment durations. This adjustment reflects our assay design, which focused on a narrow time window in which HDP protein knockdown was significant and would have caused a growth defect in the absence of yDHODH rescue. Our goal was to use chloroquine as a control to assess assay responsiveness, rather than to re-evaluate its role as a hemozoin inhibitor. Therefore, it was not necessary to precisely replicate the treatment conditions from the original publication by Combrinck et al. (2015), as long as the phenotype observed with this control behaved as expected. Given that shorter treatment durations generally increase the effective IC_{50}, we believe that the chloroquine concentration chosen falls within a reasonable and appropriate range.

Figure 5F-H: The figure legend should specify that relative hemozoin, hemoglobin, and heme refer to the absolute amounts of each species per parasite (as opposed to % amounts), normalized to the absolute amount in the DMSO-treated, no drug control. The statistical analysis used should also be included in the legend.

We have now included this information in the revised Figure legend (Lines 927-930):

*“Relative heme pools correspond to the absolute amounts of each species per parasite, normalized to the DMSO control without CQ. Mean and individual values are shown. *P<0.01; **P<0.01; one-way ANOVA with Tukey’s post hoc test; N = 3 biological replicates with 2 technical replicates each.”*

Pg. 9, line 323-325 and pg. 33 Supplementary Figure S3F: The authors state that RAP treatment sensitizes the *hdp-smHA:loxP* + *ydhodh* NF54 line to proguanil. However, the data in Supplementary Figure S3F appear to show the opposite trend. The DMSO and RAP curves may have been incorrectly labelled.

We apologize for this oversight. The DMSO and RAP groups were inadvertently mislabeled, and this has now been corrected in the revised figure.

Pg. 8, line 320: Change “haemagglutinin” to “hemagglutinin” to maintain consistency with American English throughout the manuscript.

This has been changed accordingly.

Pg. 10 Line 362, 363, and 385: Define the abbreviations GFP, Co-IP, HRP upon their first appearance.

We have removed the abbreviation for Co-IP, as it is mentioned only once in the text, and retained the full term. The abbreviations GFP and HRP are now defined at their first occurrence.

Reviewer #2

I co-reviewed this manuscript with one of the reviewers who provided the listed reports. This is part of the *Nature Communications* initiative to facilitate training in peer review and to provide appropriate recognition for Early Career Researchers who co-review manuscripts.

We again thank Reviewers #1 and #2 for their constructive feedback.

Reviewer #3

Having previously studied aspects of the *Plasmodium falciparum* (*Pf*) digestive vacuole physiology, an important antimalarial drug target, L. Sarrazin, J. Matz and colleagues turn their attention in the studies reported in this manuscript to *Pf*HDP (heme detoxification protein), thought to be critical for heme biomineralization in the digestive vacuole. Using a series of transgenic *Pf* lines, in which HDP is tagged and/or subject to conditional knockout, the authors find that (tagged) HDP is primarily found in the parasite mitochondrion rather than the previously reported locations (parasite cytoplasm and digestive vacuole and RBC cytoplasm), and depletion of *Pf*HDP did not affect hemozoin formation. HDP was, however, found to be critical for maintenance of mitochondrial electron transport and membrane polarization. Deletion of the hdp gene was lethal but lethality could be overcome by the transgenic expression of yDHODH (yeast dihydroorotate dehydrogenase), which provides an alternate path to pyrimidine biosynthesis not linked to ubiquinone and the electron transport chain (ETC). Δhdp (ydhodh+) lines were hypersensitive to proguanil, the synergistic partner drug to the ETC inhibitor atovaquone in the antimalarial Malarone. Loss of atovaquone-sensitive oxygen consumption was also observed in Δhdp (ydhodh+) parasites. Structural comparisons using AlphaFold structural predictions and experimental structures, where available, confirmed the likely ortholog of *Pf*HDP in *Toxoplasma*, TGME49_285272, which is a mitoribosomal protein (mL135) located at the LSU-SSU interface. Indeed, loss of hdp correlated with depletion of many ETC complex subunits and mitoribosomal proteins, as observed in proteomics experiments.

In general, the manuscript was well-written. The data was well presented and substantive and supported by appropriate statistical tests when appropriate. This work should be of interest to parasitologists, especially malariologists, tropical medicine and drug development scientists.

We thank Reviewer #3 for their careful evaluation and constructive feedback.

There are a few points I think should be clarified. One area concerns the construction and validation of the transgenic *Pf* lines. The sequences of two principal repair constructs are provided in the supplementary materials. First, in the *hdp-smHA:loxP* + *ydhodh* construct, there is a nonsynonymous change in the coding sequence resulting in a Phe to Leu substitution at aa position 91. Is there a rationale for this?

MKNRFYYNLIIKRLYTRSGGLRKPQKVTNDPESINRKVYWCFEHKPVKRTIINLIYSHNELKIFSNLLNHP TVGSSLIHELSLDGPYTAFL…

We thank the reviewer for bringing this to our attention. The mutant was originally intended to be generated on a Dd2 background, which carries a leucine rather than a phenylalanine at this position – a variant present in a large number of *P. falciparum* isolates, including both drug-sensitive and resistant strains. While this was an oversight on our part, we took the opportunity to evaluate the impact of this common variant on proguanil and chloroquine sensitivity, using the parental NF54$^{attB\text{-}DiCre}$ parasites as a control. As expected, the amino acid substitution had no effect on proguanil responses, although complete knockout did. Chloroquine sensitivity did not change with either the amino acid substitution or complete HDP deletion. We have now incorporated these new data into Supplementary Figure S5 and revised the corresponding section of the manuscript accordingly. Additionally, we have renamed the mutant to reflect the amino acid substitution (Lines 331-334, 337-343, and 366-368):

"Additionally, we introduced an F91L amino acid substitution into the HDP coding sequence, creating a variant commonly observed across different P. falciparum isolates. This allowed us to assess not only the effects of HDP presence versus absence, but also to account for natural sequence variation using the same parasite line."

"The hdp-F91L-smHA:loxP + ydhodh NF54 line replicated efficiently following RAP treatment and reproduced the proguanil hypersensitivity phenotype observed in the 3D7-based mutant (Supplementary Fig. S5e,f). In contrast, non-induced hdp-F91L-smHA:loxP + ydhodh NF54 parasites displayed normal proguanil sensitivity compared to the parental NF54$^{attB\text{-}DiCre}$ line, indicating that the F91L variant can functionally replace the endogenous protein. These results suggest that the role of HDP in mitochondrial protein synthesis is conserved across different strains and naturally occurring protein variants."

"Consistent with our findings in the 3D7-based line, neither the F91L substitution nor excision of the entire HDP gene altered chloroquine sensitivity in the hdp-F91L-smHA:loxP + ydhodh NF54 parasites (Supplementary Fig. S5g)."

These new data prompted us to divide the section into two individual subchapters, titled (Lines 326 and 344):

"HDP function is conserved across strains and protein variants"

"HDP does not function in heme biomineralization"

Nanopore/whole genome sequencing is part of the validation of the resulting lines, and some nanopore reads are provided in the supplementary materials. The sequence from the RAP-treated deletion line was puzzling; self-BLAST dot-plot indicated the presence of repeated sequences at the 5' end. I mapped the elements present and observed ~2100 bp at the 5' end that consists of an (imperfect) inverted repeat, which includes 2 loxP elements (a normal CRE-lox deletion would leave only a single loxP). The provided sequences do not extend into the surrounding genomic DNA, so we cannot be sure that this apparent rearrangement has not caused changes to the surrounding loci. This question should be

addressable in the whole genome sequence alluded to in the manuscript, but this data doesn't seem to be easily analyzed by the average reviewer/reader on their PC/laptop; hopefully there were no changes that would affect the results, and in that case, I just suggest that additional accessible sequence data be provided that covers the repair-genomic DNA junctions and immediately surrounding loci.

We thank the reviewer for bringing this to our attention. We are confident that the additional sequences 5' to the expected amplicons result from sequencing and assembly artefacts generated by Plasmidsaurus (which uses Oxford Nanopore technology for amplicon sequencing), rather than from repeated sequences integrated into the genome. Diagnostic PCR 6, which includes a forward primer 77 bp upstream of the construct, produces the expected band sizes both with and without RAP treatment. Please refer to the updated Supplementary Figure S5b, which now includes the same PCR reactions that were submitted for sequencing (clone 3). Notably, an insertion of 2-5 kb would be readily detectable on the agarose gel, particularly in the RAP-treated sample.

Analysis of the raw FASTQ files indicate that 100% of reads map to the construct, with a small fraction appearing to be concatemers of the original amplicon – a known artefact in Oxford Nanopore sequencing. To analyze the sequencing data, raw reads were first aligned to the construct using minimap2. The Python packages *pysam* and *ssw-align* were then used to identify soft-clipped reads (i.e., reads with 5′ and/or 3′ overhangs relative to the reference) and to determine whether subsequences within these overhangs locally align to the construct. This analysis showed that, with the rare exception of low-complexity sequences likely attributable to sequencing noise, all overhang sequences confidently map to different regions of the construct. Consistent with this, the artificial chromatogram (.ab1) files provided by Plasmidsaurus alongside the assembled FASTA show minimal support for the additional sequence. In addition to the .ab1 files, we have now included the trimmed assembly sequences and raw FASTQ files.

As suggested by the reviewer, we have revisited the whole-genome sequencing data and confirmed that no additional sequences were integrated into the genome. We also validated the DNA junctions between the repair template and the bordering genomic regions. The sequence data corresponding to the modified HDP locus and its flanking regions have been extracted from the dataset and are now available as an additional Supplementary Sequence file in FASTA format (Supplementary Data S1).

In the methods describing the generation of the 3D7-based *hdp-mNG:loxP* line, it describes "…the mNG tag was cloned in-frame using HpaI and EcoRV restriction sites." [manuscript lines 458-458], but we are not informed what plasmid contains these sites; it should be described here, along with the plasmid used to express Cas9 and the sgRNAs (to allow for reproduction of the work).

We thank the reviewer for the suggestion and agree that this section benefits from additional detail. Accordingly, we have revised the Supplementary Methods section:

> *"For generation of the 3D7-based hdp-mNG:loxP mutant, a Cas9 repair template was generated by commercial gene synthesis and provided in the pUC-GW-AMP recipient vector (Azenta Genewiz). The mNG tag was PCR-amplified, digested with HpaI and EcoRV, and cloned in-frame immediately in front of the HDP stop codon, using an HpaI site that had been incorporated during gene synthesis specifically for this purpose. Guide plasmids were generated by annealing complementary site-specific oligonucleotides and cloning them into BbsI-digested pDC2-Cas9-hDHFRyFCU, which contains both the Cas9 expression cassette and the WR99210 resistance marker[1]. Prior to transfection, the repair plasmid was linearized with AhdI, which cuts within the bacterial backbone. DiCre-expressing P. falciparum schizonts (strain B11)[2] were transfected with a mixture containing 20 µg circular guide plasmid and 60 µg linearized repair plasmid using a 4D Nucleofector system (Lonza). Transgenic parasites were selected with 10 nM WR99210 (Jacobus Pharmaceuticals)."*

In addition, some of these details are also provided in the footnotes of Supplementary Table S1:

> *"Complementary oligonucleotides were annealed, phosphorylated and cloned into the pDC2-Cas9-hDHFRyFCU plasmid using BbsI for the 3D7-based mutant. Alternatively, annealed oligonucleotides were inserted into AflII-digested pAIO-3 plasmid using In-Fusion cloning for the NF54-based mutant."*

Also the method of excising the "linearized repair template" should be described; judging by Fig. 1A, it looks like the repair template is cut out of its host plasmid without carrying along any of the plasmid in contrast to another commonly used method, in which the cut site(s) are placed between the 3' HR and 5' HR so that almost all of the plasmid, including a selectable marker, are included in the linearized construct and incorporated in the genome following repair.

As noted above, the 3D7-based repair template shown in Supplementary Table S1 was generated by commercial gene synthesis and delivered in a standard bacterial shuttle plasmid. Prior to transfection, the bacterial backbone was cleaved with a single enzyme, resulting in the repair template being flanked on both sides by fragments of the shuttle plasmid. This information is now included in the revised Supplementary Methods section:

> *"Prior to transfection, the repair plasmid was linearized with AhdI, which cuts within the bacterial backbone."*

The bacterial "overhangs" are thought to be beneficial, as they increase the in-cell stability of the repair template, protecting it from rapid degradation and allowing more time for recombination. It is also important to note that, for the 3D7-based mutant, no additional sequences are integrated into the genome. The selection cassette resides on the co-transfected episomal guide plasmid, which is rapidly lost and typically absent after clonal isolation. The overhangs are not integrated into the genome but are degraded within the parasite. For this reason, they have been omitted from the schematic as they are irrelevant to the recombination event; we now specify this more clearly in the Figure legend (Lines 835-837):

> *"Linearization of the repair template generates flanking bacterial backbone fragments, which have been omitted for clarity as they are irrelevant to recombination."*

By contrast, in the generation of the NF54-based line, the entire plasmid, including the bacterial backbone and drug cassette, is integrated into the genome, as indicated in Supplementary Table S1.

In Figure 1A diagram of the *hdp-mNG:loxP* line generation process, the position of primer P5-R is misrepresented. P4-R should be about where P5-R is shown (well downstream of the 3'HR), and P5-R should be close to the middle of the 3'HR. The position of the primers is relevant as it allows the reader to see which PCR reactions verify the insertion-genomic DNA junctions and which report elements internal to the repair construct inserts. Similarly, in Fig S3A, primer P6-R should be shown downstream of the 3'UTR.

We thank the reviewer for pointing this out. We have updated the Figure panels to more accurately depict the position of each primer relative to the homology regions.

Additionally, The P3-R and P4-F primer binding sequences are located in the *loxP* elements, i.e., they each occur 2X in the repair templates. Is there a reason for this, and one wonders why only the shorter of the possible PCR products appears in each case? If the PCR conditions were adjusted to favor short products, this should be described in the Methods section.

It is correct that the *loxP* primers anneal at two sites, potentially generating two products for each integration-specific PCR (PCR3: 807 vs. 2,026 bp; PCR4: 1,593 vs. 2,812 bp). Although

we did not optimize conditions to favor shorter products, *e.g.* by using shorter elongation times, we consistently observed only the smaller amplicon. This observation is entirely consistent with PCR kinetics. DNA polymerases extend shorter fragments more quickly and reliably than longer ones, and because PCR amplification is exponential, this early advantage is progressively amplified as the short and long products compete for the same primers. Consequently, the longer fragment has fewer effective cycles to accumulate before the shorter fragment dominates. This is now noted in the revised Figure legend (Lines 838-839):

> *"Although loxP primers anneal twice, only the smaller product is observed due to preferential amplification."*

The *loxP* site was used for these PCRs because the HDP sequence in the repair template remains similar to the original, even after recodonization. In our previous work with unrelated DiCre mutants, we observed that primers targeting the recodonized gene often cross-reacted with the original locus. Using *loxP*-specific primers avoids this issue, ensuring amplification is specific to the intended target.

Other minor suggestions/questions follow:

Lines 93-95: The question of reaction volume and reaction essentiality are not necessarily directly linked. Are there actual measurements on this question? In the absence of metabolic flux measurements of the relative reaction mass processed by each quinone-dependent dehydrogenase in wild type blood stage parasites during normal growth, one cannot say whether one dehydrogenase donates significantly more electrons to the ETC than each or all of the others. If this is the case, then statements about minor and main electron donating enzyme reactions should be removed.

We thank the reviewer for this insightful comment. Indeed, we could not find any studies directly quantifying respiratory electron flow in malaria parasites. Accordingly, we have rephrased the section to avoid referring to the electron contribution of individual dehydrogenases (Lines 104-107):

> *"~~This suggests that their contribution to feeding electrons into complex III is minor. The main enzyme donating electrons to~~ Complex III further receives electrons from ~~is believed to be~~ dihydroorotate dehydrogenase (DHODH), which produces the pyrimidine precursor orotate and transfers the electrons to ubiquinone (10)."*

Lines 121-122: Recently transposon screens of *P. knowlesi* were reported. Elsworth et al (2025) had particularly dense coverage and also found that the HDP ortholog was likely essential in Pkn. If desired, this could be cited here to increase the range of spp. in which HDP appears to be essential. [Elsworth B, Ye S, Dass S, Tennessen JA, Sultana Q, Thommen BT, Paul AS, Kanjee U, Grüring C, Ferreira MU, Gubbels MJ, Zarringhalam K, Duraisingh. The essential genome of *Plasmodium knowlesi* reveals determinants of antimalarial susceptibility. MT.Science. 2025;387(6734):eadq6241. doi: 10.1126/science.adq6241.]

This reference has now been added to the manuscript.

Line 133: "…conflicting localizations for HDP," Under the previous hypothesis for trafficking of HDP to the DV these were thought to be sequential steps of the pathway, as the authors here discuss elsewhere in the manuscript. I suggest using phrasing like "multiple localizations".

This has been revised according to the reviewer's recommendation (Lines 144-145):

> *"Previous studies have reported ~~conflicting~~ multiple localizations for HDP, placing the protein in the RBC cytosol, the DV, and elongated organelles within the parasite[33,37]."*

Lines 135-136 and Fig. 1: Note that the mNG tag is larger than HDP (the smHA tag is even larger).

Large tags have been known to sometimes cause mislocalization; this caveat should be mentioned (and discounted if appropriate). Since the localization of HDP shown here differs from that reported previously, possible reasons for the differences should be discussed somewhere in this manuscript.

We thank the reviewer for this insightful comment. We now include statements about the tag sizes in the legends of Figure S5 and Figure 1 (Line 835):

"Please note that the spaghetti monster hemagglutinin tag (smHA) is larger than depicted."

"Please note that the mNG tag is larger than depicted."

Additionally, we now critically discuss the potential for mislocalization induced by the tags (Lines 405-409):

"The mNG and smHA tags used in this work are relatively large, raising the possibility of functional interference. However, because HDP is essential for parasite survival, the ability to fuse the endogenous protein to these tags without compromising viability indicates that the observed localization reflects the physiological distribution of HDP."

We also revisited whether a small secondary fraction of HDP-mNG might localize to the RBC cytosol or the DV, as previously suggested. First, we reanalyzed our previous images of B11 and *hdp-mNG:loxP* parasites and quantified the signal in the host cell compartment, revealing no difference between the lines and indicating that the protein is not exported. These new data are now presented in an additional Supplementary Figure S1 and discussed in the revised Results section of our manuscript (Lines 156-158):

"Similarly, no signal was detected within the host cell compartment, indicating that the protein is not exported, contrary to previous suggestions (Fig. 1c, Supplementary Fig. S1a)[33,37]."

We then considered that the mNG tag might be quenched within the DV due to the acidic environment. We therefore quantified the green fluorescent vacuolar signal following treatment with the ionophore nigericin or the V-ATPase inhibitor concanamycin A, both of which increase vacuolar pH, but again observed no difference between B11 and *hdp-mNG:loxP* parasites. To confirm that these treatments effectively deacidified the DV, we applied them to a previously described biosensor line in which the pH-sensitive GFP derivative superecliptic pHluorin is targeted to the DV, where it is normally non-fluorescent due to acid quenching (Alder et al., 2023). Both nigericin and concanamycin A induced robust DV fluorescence in this line, demonstrating that the treatments successfully raised vacuolar pH. These data are now included in the new Supplementary Figure S1 and are discussed in the revised Results section (Lines 158-164):

"To exclude the possibility that the mNG signal is quenched in the DV due to low pH, we treated parasites with the V-ATPase inhibitor concanamycin A or the ionophore nigericin, both of which deacidify the vacuolar lumen. Under these conditions, we again observed no difference in DV signal intensity between B11 and hdp-mNG:loxP parasites (Supplementary Fig. S1b-d). In contrast, the same treatment markedly increased vacuolar fluorescence in a previously published mutant expressing the pH-sensitive green fluorescent protein (GFP) derivative superecliptic pHluorin in the DV (Supplementary Fig. S1e)[40]."

The Methods section has been updated accordingly (Lines 560-563):

"To assess the impact of vacuolar pH on DV-localized signals, parasites were treated for 15 minutes with 50 mM nigericin or for 30 minutes with 61 nM concanamycin A

prior to imaging. As a positive control, we included the previously published pm2-SEP mutant, which expresses the pH biosensor superecliptic pHluorin in the DV[40].”

Together, these new findings strongly support our previous conclusion that HDP localizes to the mitochondrion and is neither exported nor trafficked to the DV. Nonetheless, we acknowledge that we cannot formally exclude the possibility that a very small fraction of HDP, undetectable by our methods, may localize to other compartments or reside there only transiently (Lines 413-414):

“We cannot entirely exclude that a very small fraction of HDP resides in, or transiently traffics through, the DV.”

In the revised Discussion, we now emphasize that the discrepancies in localization reported in earlier studies and our own largely depend on whether antibodies or fluorescent protein tags were used. While we are confident that mitochondrial localization is physiological and suspect that non-specific antibody labeling may have contributed to earlier observations, we avoid discrediting previous work, leaving interpretation of these conflicting data to the reader. Instead, we subtly point toward a plausible explanation without explicitly suggesting flaws or limitations in prior studies (Lines 410-413):

“It is worth noting that the reported subcellular distribution of HDP in previous studies largely depended on the method of detection, with antibody-based approaches consistently suggesting export and reuptake, whereas fluorescent protein tagging indicated organellar localization within the parasite[33,37].”

In Fig 2 H& I, the meaning of the data bar colors in the graphs should be described in the caption.

For improved clarity, a color key has been added to panel 2h; Panel 2i already included a key.

Fig 3J. The authors should probably quantitate the relative band intensities using software such as ImageJ or Fiji.

We performed densitometric analysis on the blots, as suggested, and the relative band intensities are now reported in the revised Figure legend (Lines 885-886):

“Densitometry shows that RAP treatment lowers NDUFA4 and Cox5B levels to 6% and 38% of control, respectively.”

We would like to clarify that in the revised Figure panel, different blots are now presented. In the original experiments, mCh and BiP antibody stainings were performed in parallel but on separate blots. To comply with journal requirements, we have now repeated the experiments and probed the same membrane sequentially, first with BiP antibodies and then, after membrane stripping, with mCh antibodies.

The only exception is the Western blot shown in Figure 2a, where the loading control is still presented on a separate blot. This was necessary because HDP-mNG was consistently restricted to the detergent-resistant pellet fraction, whereas the loading control protein is fully solubilized under these conditions. Consequently, the pellet fraction was used for detection of HDP-mNG, while the soluble fraction was used for probing BiP. This clarification has now also been included in the figure legend (Lines 854-855):

“Note that mNG and BiP were probed on separate blots, using the detergent-resistant membrane pellet for mNG and the soluble fraction for BiP, respectively.”

The protocol used for membrane stripping is now mentioned in the revised Methods section (Lines 519-521):

“Sequential labeling of the same blots with different antibodies was achieved by including a stripping step between incubations, in which blots were treated with 200 mM glycine and 0.1% SDS at pH 2.2.”

Fig. 4A. The shading of the numbers in the alignment is faint, making it hard to perceive colors mentioned in the caption. Perhaps these could be darker.

This has been changed accordingly.

Lines 265-278 and Fig. 4 examine the sequence and, especially, structural similarities of *Pf*HDP to Fas1 domain proteins in *Toxoplasma gondii*, concluding that TGME49_285272 (mL135 in Shikha et al mitoribosome structure) is the likely ortholog of *Pf*HDP. If *Pf*HDP is an important mitoribosomal protein conserved in *Toxoplasma* and *Plasmodium*, which are not the closest evolutionary “cousins”, one would expect orthologues in other Apicomplexan species with mitochondria and perhaps other in related groups. It would be of interest, then, to determine the extent of such putative orthologues in Apicomplexa and related phyla. A few quick BLAST searches found such apparent orthologues in *Theileria*, *Babesia*, *Chromera* and *Vitrella* spp., for example.

TA11080 hypothetical protein, conserved (with C-terminal Fas1 domain)
BBOV_III009730 heme detoxification protein (fasciclin I)
Cvel_24500 Heme ligase, putative (has C-terminal Fas1 domain)
Vbra_10965 Heme ligase, putative (has C-terminal Fas1 domain)

This is indeed an interesting area, which we have explored further in the revised manuscript. We identified putative orthologs of HDP in the related piroplasmid parasites *Babesia* and *Theileria*, as well as in more distant relatives, including additional coccidians and in the chrompodellid clade. Notably, none of these parasites generate hemozoin, yet they appear to retain HDP, casting further doubt on its previously proposed function in heme biomineralization. Intriguingly, we found no orthologs in *Cryptosporidium* species, which have lost their mitochondrial genome along with the mitochondrial translation apparatus, providing additional support for a role of HDP in the mitoribosome.

We now include a phylogenetic tree and an identity matrix for the included proteins as an additional Supplementary Figure S7 and comment on these results in the revised Discussion section (Lines 416-420):

“Importantly, genome database searches reveal likely HDP orthologs in closely related piroplasmid parasites, such as Babesia and Theileria, which do not form hemozoin, and show a lack of clear orthologs in Cryptosporidium species, where the mitochondrial genome and mitoribosome have been lost (Supplementary Fig. S7).”

Fig. 4E. pLDDT should be defined/described.

This has been included in the legends of Figure 4 and Supplementary Figure S3.

In line 463, “In-Fusion cloning” should be described or referenced.

The kit used for cloning is now referenced in the revised Supplementary Methods section:

“For the hdp-F91L-smHA:loxP + ydhodh NF54 line, the repair plasmid was generated by assembly PCR and introduced into XhoI/EagI-digested yPM2GT vector, which carries a yDHODH drug-selectable marker[3], using In-Fusion cloning according to the manufacturer’s instructions (Takara Bio; 638955).”

We would like to thank the reviewer again for their constructive feedback and generous time commitment.

Reviewer #4

The paper titled "Redefining the role of the *Plasmodium* heme detoxification protein: From hemozoin formation to mitochondrial protein synthesis," by Sarrazin et al., aims to address an interesting and long-standing question in malaria biology: the function and localization of the heme detoxification protein (HDP). The paper is well-written, clearly structured, and the experiments were performed rigorously, offering an interesting new perspective on HDP biology that is sure to interest scientists working on various aspects of hemoglobin metabolism, hemozoin biosynthesis, and parasite organellar biology.

We thank Reviewer #4 for their careful evaluation and constructive feedback.

What is interesting about this paper is the attempt to revisit the traditional model of hemozoin biosynthesis, in which HDP is known to play a role. The section on HDP localization, however, could benefit from integrating the earlier work on this topic to define current thinking on heme detoxification in *Plasmodium falciparum*. The original work by Jani et al. (2008) identified HDP as a heme-binding protein that promotes β-hematin formation *in vitro*. In that study, the authors proposed an unusual trafficking pathway in which HDP is exported to the cytosol of the infected erythrocyte and subsequently re-internalized during hemoglobin endocytosis, eventually accumulating in the digestive vacuole. Based on these observations, HDP was suggested to participate directly in detoxifying free heme released during hemoglobin degradation. Subsequent work has refined the mechanistic understanding of hemozoin formation. In particular, Matz et al. (2020) demonstrated that the digestive-vacuole protein PV5, a lipocalin-like protein, plays a regulatory role in hemozoin crystal growth and organization. Rather than directly catalyzing heme crystallization, PV5 appears to guide the ordered assembly of hemozoin within the digestive vacuole. These findings suggested that hemozoin biomineralization may be controlled by specialized vacuolar proteins and structural regulators, rather than relying exclusively on HDP-mediated catalysis.

Major points:

The detection of HDP in the erythrocytic cytosol following streptolysin-O-induced permeabilization should be interpreted with caution. This treatment can disrupt parasite integrity and release intracellular organelles, potentially exposing parasite proteins outside their native compartments.

We thank the reviewer for highlighting this previous work. Gupta et al. (2022) fractionated infected RBCs using the pore-forming toxin streptolysin O to selectively lyse the host cell membrane. Western blot analysis showed that, when expressed as a GFP fusion, most HDP remained associated with the parasite pellet, with strong bands at ~50 kDa (likely the full fusion protein) and ~25 kDa (corresponding to GFP alone). Only a tiny fraction of the GFP band was detected in the supernatant, indicating that the vast majority – if not all – of the protein is retained within the parasite. In stark contrast, antiserum raised against HDP recognized the endogenous protein much more prominently in the supernatant fraction.

Similar discrepancies were observed in imaging-based approaches reported by Gupta et al. (2022). Their live microscopy of HDP-GFP clearly localized the protein to an elongated parasite organelle that branches as soon as parasites enter schizogony, highly reminiscent of the mitochondrion, with no detectable GFP signal in the DV or RBC cytosol. By contrast, immunofluorescence using HDP antisera showed numerous puncta both inside and outside the parasite, partially overlapping with hemoglobin.

In the original study characterizing HDP, Jani et al. (2008) also reported conflicting localizations that depended on the detection method: antibody labeling suggested abundant export, whereas YFP tagging indicated retention within the parasite with distinct accumulations. Combined, this may suggest potential cross-reactivity of the HDP antibodies, possibly with host cell antigens, which could explain why such a large fraction of HDP was previously reported to associate with the hemoglobin trafficking pathway.

In the revised Discussion, we now highlight that the observed discrepancies in localization largely depend on whether antibodies or fluorescent protein tags are used (Lines 410-413):

> *"It is worth noting that the reported subcellular distribution of HDP in previous studies largely depended on the method of detection, with antibody-based approaches consistently suggesting export and reuptake, whereas fluorescent protein tagging indicated organellar localization within the parasite[33,37]."*

To further address the possibility of RBC cytosol localization, we reanalyzed our previous images by measuring signal intensities in the cytoplasm of both B11- and *hdp-mNG:loxP*-infected RBCs. No differences were observed, with signal intensities falling within the range of background noise, indicating that HDP-mNG is not trafficked through the RBC cytosol. These data are now included in a new Supplementary Figure S1 and highlighted in the revised Results section (Lines 156-158):

> *"Similarly, no signal was detected within the host cell compartment, indicating that the protein is not exported, contrary to previous suggestions (Fig. 1c, Supplementary Fig. S1a)[33,37]."*

The methods section has been updated accordingly (Lines 557-559):

> *"Signals in the DV and RBC cytosol were quantified at 24 hours post-invasion after manually outlining the compartments."*

Although the mNG and smHA tags used in our study are quite large, we consider it unlikely that they interfere with HDP trafficking or function, as addressed in our revised Discussion section (Lines 405-409):

> *"The mNG and smHA tags used in this work are relatively large, raising the possibility of functional interference. However, because HDP is essential for parasite survival, the ability to fuse the endogenous protein to these tags without compromising viability indicates that the observed localization reflects the physiological distribution of HDP."*

At the same time, the absence of a strong HDP signal in the digestive vacuole when the protein is fused to mNeonGreen does not necessarily exclude a transient localization in this compartment. Fluorescent proteins often lose signal in acidic environments, such as the digestive vacuole, complicating the interpretation of fluorescence-based localization experiments. Therefore, it remains possible that HDP transiently traffics through the digestive vacuole or participates in a shuttle-type pathway linked to hemoglobin processing.

We thank the reviewer for raising this important point. It is correct that fluorescent proteins can be pH-sensitive and lose fluorescence under acidic conditions such as those in the DV (pH~5). A clear example is superecliptic pHluorin, a GFP derivative with a pKa of 7.0, which is almost completely non-fluorescent in the DV, as we have previously shown (Alder et al., 2023). With a pKa of 5.7, mNG is considered moderately acid-sensitive, suggesting that the low pH within the DV could potentially have influenced our previous observations.

To address this, we performed additional quantitative imaging experiments under conditions that deacidify the vacuolar matrix, using the V-ATPase inhibitor concanamycin A and the ionophore nigericin. Using these treatments, we compared green fluorescence in the DV of parental B11 parasites and the *hdp-mNG:loxP* mutant and again detected no differences in signal intensity. Effective DV deacidification was confirmed using a positive control line expressing superecliptic pHluorin fused to the DV protease plasmepsin 2, which showed the expected increase in fluorescence.

These findings have been included in the new Supplementary Figure S1 and are now discussed in the revised manuscript (Lines 158-164 and 560-563):

> *"To exclude the possibility that the mNG signal is quenched in the DV due to low pH, we treated parasites with the V-ATPase inhibitor concanamycin A or the ionophore nigericin, both of which deacidify the vacuolar lumen. Under these conditions, we again observed no difference in DV signal intensity between B11 and hdp-mNG:loxP parasites (Supplementary Fig. S1b-d). In contrast, the same treatment markedly increased vacuolar fluorescence in a previously published mutant expressing the pH-sensitive green fluorescent protein (GFP) derivative superecliptic pHluorin in the DV (Supplementary Fig. S1e)[40]."*

> *"To assess the impact of vacuolar pH on DV-localized signals, parasites were treated for 15 minutes with 50 mM nigericin or for 30 minutes with 61 nM concanamycin A prior to imaging. As a positive control, we included the previously published pm2-SEP mutant, which expresses the pH biosensor superecliptic pHluorin in the DV[40]."*

In addition, we provide evidence that HDP, when overexpressed as an mCh fusion protein, still shows no DV signal. With a pKa of 4.5, mCh fluorescence is highly resistant to acidic conditions. We have highlighted this point in the revised manuscript (Lines 164-167):

> *"To further examine a potential DV localization, we overexpressed HDP fused to the acid-resistant red fluorescent protein mCherry (mCh), but despite the much stronger fluorescence, localization remained restricted to the branching organelle, with no apparent signal in the DV (Fig. 1e)."*

To further clarify this issue, complementary biochemical approaches could be extremely informative. For example, isolating parasite mitochondria (https://doi.org/10.1038/nature05572) and digestive vacuoles (https://doi.org/10.1016/S0006-2952(98)00140-3), followed by immunoblotting with anti-HDP antibodies, could provide direct evidence of HDP enrichment in specific organellar fractions through the parasite cell cycle. Such experiments would help clarify the current interpretation in relation to previous studies and strengthen the conclusions regarding HDP localization.

We agree with the reviewer that comparison with orthogonal experimental approaches would be beneficial. Fortunately, several independent data sets support our observations: (1) Proteome analysis of purified DVs recovered many known vacuolar proteins, including V-ATPase subunits, the lipocalin PV5, and hemoglobin-specific proteases, but did not detect HDP (Lamarque et al., 2008). (2) HyperLOPIT clearly identified the *Toxoplasma* HDP ortholog TGME49_285272 in the mitochondrion (Barylyuk et al., 2020). (3) Integration of multiple omics datasets has been used to predict mitochondrial localization, with HDP ranking highly in these predictions (van Esfeld et al., 2021). (4) Genome database searches reveal likely HDP orthologs in *Babesia* and *Theileria*, which do not form hemozoin, but not in *Cryptosporidium*, where the mitochondrial genome and mitoribosome have been lost.

In the revised manuscript, we now discuss our observations in the context of these data (Lines 414-420):

> *"However, HDP was not detected in the proteome of purified DVs[57] and instead emerged as a high-ranking candidate in datasets predicting mitochondrial localization based on integrated omics analyses[58], consistent with our observations of its subcellular distribution. Importantly, genome database searches reveal likely HDP orthologs in closely related piroplasmid parasites, such as Babesia and Theileria, which do not form hemozoin, and show a lack of clear orthologs in Cryptosporidium species, where the mitochondrial genome and mitoribosome have been lost (Supplementary Fig. S7)."*

As the reviewer noted with respect to streptolysin O, fractionation approaches are not trivial. DV preparations have historically been contaminated with material from the ER, nucleus, and mitochondrion (e.g., Lamarque et al., 2008; PMID: 21136929). Similarly, mitochondrial fractions frequently contain proteins from other compartments, including the apicoplast, and, importantly, the DV (e.g., Kobayashi et al., 2007; PMID: 17289446). These isolations should therefore be considered enrichment procedures rather than true purifications. Consequently, the mixed proteome of these preparations complicates the interpretation of HDP-specific bands in Western blots derived from such fractions. We believe the strength of our approach lies in avoiding these pitfalls by preserving the cellular context and analyzing the protein in its native biological environment.

We assessed HDP localization throughout the entire asexual cycle and consistently detected the protein only in the mitochondrion (Figure 1c), an observation further supported by our new quantitative imaging data sets (Supplementary Figure S1). At the same time, we agree with the reviewer that a minor fraction of HDP may still reside in the DV, or that DV residency could be transient or represent a trafficking intermediate. We now acknowledge this possibility in the revised manuscript (Lines 413-414):

> *"We cannot entirely exclude that a very small fraction of HDP resides in, or transiently traffics through, the DV."*

Together with the revisions to the manuscript, we are confident that our work robustly supports our claims. We employ a combination of complementary approaches, including (1) quantitative microscopy, (2) mitochondrial bioenergetics, (3) quantitative heme analyses, (4) structural modelling, (5) unbiased proteomics, and (6) gene orthology analysis – all of which consistently point to mitochondrial functions and argue against a role in heme biomineralization. Importantly, we wish to re-emphasize that rescuing the respiratory deficiencies in HDP-null parasites restores both normal growth and efficient hemozoin formation, demonstrating that the protein's essentiality is attributable solely to its role in mitochondrial electron transport. We therefore believe, that the optimization and implementation of the proposed fractionation experiments – particularly in a time-resolved manner and given the inherent contamination of such preparations – lie beyond the scope of this revision.

It would also be valuable for the authors to review the following work on mitochondrial biology in *Plasmodium falciparum*. Scarpelli et al. (2018) showed that mitochondrial dynamics in the parasite are regulated through fission-related proteins, highlighting the importance of mitochondrial homeostasis for parasite survival and development. More recently, Sparkes, Penny C., et al. (2024) examined mitochondrial function during gametocytogenesis and demonstrated that mitochondrial activity and ATP production are important for parasite differentiation. Although this study focuses on sexual-stage development, it shows the mitochondrion's central role in coordinating key cellular processes in the parasite. These studies collectively support the view that the parasite mitochondrion functions within a broader cellular network rather than as an isolated organelle. In this light, discussing this literature could strengthen the possibility that HDP localization to mitochondria reflects a previously unrecognized connection between mitochondrial function and pathways related to hemoglobin metabolism and heme detoxification.

We thank the reviewer for highlighting this interesting body of literature, which has undoubtedly contributed to our understanding of mitochondrial biology in *Plasmodium* and underscores the broader cellular context in which this organelle operates. While these studies are valuable, we do not believe they provide direct support for a connection between mitochondrial function and heme detoxification. Given the need to limit the number of references, we would therefore prefer not to include this literature in the present context.

In the revised manuscript, we carefully acknowledge the previously published localizations and functional data, and we also include new experiments to re-evaluate the potential localization of HDP to the DV and the RBC cytosol. Nonetheless, our findings remain in clear contrast to these prior observations. While differences in experimental approaches might contribute to these

discrepancies, our data consistently indicate that HDP does not localize to the DV or RBC cytosol and does not participate in heme biomineralization. Rather than explicitly critiquing earlier methodologies, we have aimed to discuss our data transparently alongside previous work, allowing readers to interpret the findings in context.

Overall, this manuscript presents a stimulating reinterpretation of HDP function and opens new perspectives on the relationship between hemoglobin metabolism, heme detoxification, and parasite organellar biology. With a more comprehensive discussion of earlier literature and additional consideration of alternative interpretations of HDP localization, the study will make an important contribution to malaria parasite cell biology and to the ongoing debate over the molecular mechanisms underlying hemozoin formation.

Once again, we sincerely thank the reviewer for their thoughtful critique and the time they invested in reviewing our manuscript. While our findings do not align with the previously proposed roles of HDP, we see this as an interesting opportunity for future discussion, perhaps best explored in a dedicated opinion piece.

Additional changes:

We noted an error in the legend of Figure 1d: the quantitative imaging was performed 24 hours post-invasion, not 36 hours. This has been corrected in the revised manuscript.

The Oxford Nanopore sequencing results, previously included in the Supplementary Information, are now explicitly cited as Supplementary Data in the revised manuscript (Lines 335-337):

> *"Correct genome editing and RAP-induced HDP loss were confirmed by diagnostic PCR, Oxford Nanopore long-read sequencing, whole-genome sequencing, and Western blot (Supplementary Fig. S5b-d, Supplementary Data S1)."*

While revising the manuscript, we realized that the incorrect publication had been cited for the pNMD3:Mito-mCherry-DHODH plasmid. This has been corrected in the revised manuscript.

We have reformatted the Figures and their legends to comply with the journal's requirements. We also used color-blind-friendly coloring in our fluorescence micrographs.

We have now added a dedicated Competing Interests section to the revised manuscript.

We have revised the title to comply with the 15-word limit (Lines 2-3):

> *"Redefining the role of the Plasmodium heme detoxification protein: From hemozoin ~~formation~~ to mitochondrial protein synthesis"*

We have revised some subheading of the results section to comply with the character limit (Lines 196-197, 213, and 228):

> *~~"Loss of hemozoin formation coincides with developmental arrest in HDP-null parasites~~ HDP loss abolishes hemozoin formation only upon parasite death"*

> *"HDP is critical for $\Delta\Psi_{mt}$ maintenance ~~of the mitochondrial membrane potential~~"*

> *"A metabolic ~~respiratory chain~~ ETC bypass rescues HDP-deficient parasites"*

To comply with the reference limit, we had to remove some citations previously included in our manuscript. When discussing broader scientific background, we now cite review articles instead of multiple individual studies.

We moved details on vector construction and genetic strategies to the Supplementary Methods section, as they are not essential for understanding the main findings of the work.

We have revised the abstract to comply with the 150-word limit.

Response to reviewers

Reviewer #1

The authors have addressed the concerns raised in the initial review. They have provided additional methodological detail and justified their drug treatment regimen for the heme fractionation assay. They have also corrected errors identified in the figures, included molecular weight markers for all gels, added explanations where requested, and ensured that all abbreviations are now properly defined upon first use.

I would prefer that the word "formation" is kept in the title to read: “Redefining the role of the Plasmodium heme detoxification protein: From hemozoin formation to mitochondrial protein synthesis.” It doesn't really make sense without the word "formation". Otherwise, I support acceptance of this manuscript for publication in Nature Communications.

We thank Reviewer #1 for their careful evaluation and constructive feedback. The manuscript title had to be shortened to comply with journal guidelines and now reads: “The *Plasmodium* heme detoxification protein functions in mitochondrial protein synthesis”

Reviewer #2

I co-reviewed this manuscript with one of the reviewers who provided the listed reports. This is part of the Nature Communications initiative to facilitate training in peer review and to provide appropriate recognition for Early Career Researchers who co-review manuscripts.

We thank Reviewer #2 for their careful evaluation and constructive feedback.

Reviewer #4

The authors have performed the additional experiments requested and have satisfactorily addressed all of my comments and queries. I am therefore satisfied with the revisions made and believe that the manuscript has been significantly improved. In its current form, I support its publication.

We thank Reviewer #4 for their careful evaluation and constructive feedback.
